# Discovery of Novel Inhibitors of *Aspergillus fumigatus* DHODH via Virtual Screening, MD Simulation, and In Vitro Activity Assay

**DOI:** 10.3390/molecules30122607

**Published:** 2025-06-16

**Authors:** Kaige Li, Wei Xia, John Z. H. Zhang

**Affiliations:** 1 Shanghai Engineering Research Center of Molecular Therapeutics and New Drug, Development, Shanghai Key Laboratory of Green Chemistry & Chemical Process, School of Chemistry and Molecular Engineering, East China Normal University, Shanghai 200062, China; 51254300105@stu.ecnu.edu.cn; 2 NYU-ECNU Center for Computational Chemistry and Shanghai Frontiers Science Center of AI and DL, New York University Shanghai, Shanghai 200126, China; 3 Department of Chemistry, New York University, New York, NY 10003, USA; 4 Faculty of Synthetic Biology, Shenzhen University of Advanced Technology, Shenzhen 518107, China; 5 State Key Laboratory of Quantitative Synthetic Biology, Shenzhen Institute of Synthetic Biology, Shenzhen Institute of Advanced Technology, Chinese Academy of Sciences, Shenzhen 518055, China

**Keywords:** AfDHODH, small molecule inhibitors, virtual screening, computer-aided drug design, binding free energy

## Abstract

To address the surge in *Aspergillus fumigatus* infections among immunosuppressed patients and azole resistance, this study focused on developing novel inhibitors targeting dihydroorotate dehydrogenase (AfDHODH), a key enzyme in fungal pyrimidine synthesis. The three-dimensional structure of AfDHODH was constructed via homology modeling. Molecular docking, dynamics simulations, and binding free energy calculations systematically elucidated the mechanisms of existing inhibitors. Virtual screening against the ZINC20 and ChEMBL databases yielded 13 candidates, with two micromolar inhibitors (IC_50_ < 100 μM) identified through in vitro assays. These inhibitors exhibited novel scaffold structures that were distinct from known DHODH inhibitors. The results validate the feasibility of homology modeling-guided antifungal discovery and these findings provide critical insights for the development of new antifungal agents.

## 1. Introduction

The clinical prevention and control of invasive fungal infections (IFIs) are facing severe challenges. Deep-seated infections caused by opportunistic pathogens such as *Aspergillus fumigatus* have shown a significant increase in incidence after the COVID-19 pandemic, becoming a major challenge in global public health [1,2,3]. Traditional antifungal drugs, including azoles (fluconazole [4], itraconazole [5], etc.), echinocandins (caspofungin [6]), polyenes (amphotericin B [7]), and flucytosine [8], have exhibited a marked decline in clinical efficacy due to the evolution of fungal drug resistance, the expansion of immunocompromised populations, and the misuse of broad-spectrum antibiotics [9,10]. Under these circumstances, the development of novel antifungal agents targeting drug-resistant fungal infections like *A. fumigatus* has become an urgent global need in infectious disease research [11].

To overcome the azole resistance problem of existing azole drugs, a new target gaining attention is *A. fumigatus* dihydroorotate dehydrogenase (AfDHODH), a key rate-limiting enzyme in the fungal pyrimidine biosynthesis pathway [12]. This oxidoreductase catalyzes the fourth step of de novo pyrimidine synthesis—the oxidation of dihydroorotate (DHO) to orotate—directly regulating the production of uridine monophosphate (UMP), an essential precursor for RNA/DNA synthesis and cell wall formation. The lethality of pyrimidine metabolism inhibition to fungal growth, coupled with the low sequence homology (approximately 30%) between fungal and mammalian DHODH, makes AfDHODH an ideal target for selective antifungal intervention [13]. Currently, the pyrrole-based small-molecule compound Olorofim, developed by F2G Ltd., has attracted significant attention for its innovative mechanism of action and has entered Phase III clinical trials [14]. By specifically inhibiting AfDHODH (IC_50_ = 44 ± 10 nM), Olorofim effectively blocks fungal DNA/RNA synthesis and demonstrates potent in vitro activity against *A. fumigatus* and various filamentous fungi [15,16]. Notably, although human DHODH operates through a similar pathway, Olorofim exhibits no detectable toxicity toward the human enzyme in vitro, owing to low homology and structural differences^14^, suggesting potential clinical safety advantages. However, challenges inherent to traditional drug development—such as lengthy trial cycles, high costs, difficulties in rapidly screening ultra-large molecular libraries for lead compounds, and the lack of crystal structures—remain obstacles to advancing virtual screening and drug discovery efforts.

The advancement of the computer-aided drug design (CADD) technology aided by the recent development of protein structure prediction methods such as AlphaFold2 [17] has provided a new paradigm for antifungal drug discovery. Integrated computational platforms incorporating homology modeling, molecular docking, virtual screening (VS), virtual high-throughput screening (vHTS), the quantitative structure–activity relationship (QSAR), and three-dimensional (3D) pharmacophore modeling have successfully achieved synergistic advantages with experimental high-throughput screening techniques, significantly accelerating the lead compound discovery process [18]. Given the unresolved crystal structure of AfDHODH, this study employed AlphaFold2 [17] to construct a homology-modeled structure of AfDHODH. Combined with molecular docking to simulate receptor–ligand binding modes, the complex structure of AfDHODH with the Olorofim molecule was predicted. To further investigate the dynamic binding mechanisms between AfDHODH and existing inhibitors, we performed nanosecond-scale molecular dynamics (MD) simulations using AMBER18 [19,20,21,22,23]. In CADD, binding free energy calculations are commonly used to evaluate ligand stability by comparing computational results with experimental values. Although free energy perturbation (FEP) and thermodynamic integration (TI) are rigorous methods for calculating binding free energy, their high computational demands and time costs limit their application in large-scale virtual screening [24,25]. In contrast, the Molecular Mechanics/Generalized Born Surface Area (MM/GBSA) method combined with alanine scanning [26,27,28,29] is widely adopted in virtual screening due to its efficiency and low computational cost, despite the frequent omission of entropy contributions owing to poor correlation and residual computational expenses [30,31]. However, the interaction entropy (IE) method developed by Duan et al. effectively addresses this limitation, demonstrating strong correlation with experimental values in other systems [32,33]. Therefore, this study utilized the IE approach for binding free energy calculations.

Using these methods, this study analyzed the interaction patterns between AfDHODH and small molecules such as Olorofim, identified the binding pocket and key residues involved in AfDHODH binding, and conducted receptor-based and ligand-based virtual screenings. The screened molecules underwent molecular dynamics simulations and binding free energy calculations. Among them, 13 commercially available small molecules were tested via in vitro enzymatic activity assays, revealing two compounds—CHEMBL1509241 and ZINC67300323—with micromolar-level inhibitory activity, exhibiting IC_50_ values of 16.86 μM and 57.33 μM, respectively. These compounds, selected through receptor-based virtual screening, feature distinct scaffolds compared to Olorofim. These findings aim to provide theoretical foundations and efficient, reliable computational methodologies for the design of antifungal drugs targeting AfDHODH.

## 2. Result and Discussion

### 2.1. Target Protein Structure Validation and Model Optimization

Using the AlphaFold2 protein structure prediction algorithm, we obtained five three-dimensional conformational models of *Aspergillus fumigatus* dihydroorotate dehydrogenase (AfDHODH) along with predicted Local Distance Difference Test (pLDDT) confidence scores (Figure S1). Among these, models rank_1 to rank_5 exhibited notably low confidence in regions spanning residues 1–80 and 435–474, likely due to the absence of experimentally resolved crystal structures for DHODH proteins with similar sequences in these regions. In contrast, most residues in other regions showed confidence scores above 90%. Notably, rank_1 demonstrated relatively higher confidence in the binding pocket region (residues 96–128), indicating superior structural prediction reliability compared to other models. Thus, the rank_1 structure was selected for subsequent complex model construction and molecular docking studies.

Previous studies [14] revealed through sequence analysis that the N-terminal residues 1–88 primarily form transmembrane domains and mediate mitochondrial targeting. Truncation of these 88 residues does not affect small-molecule inhibitor (e.g., Olorofim) binding or enzymatic catalytic activity. For the low-confidence loop region (residues 435–474), which is distant from the active site and lacks direct interaction evidence, this domain was excluded in subsequent molecular dynamics simulations to focus on catalytically functional regions.

**Figure 1:**
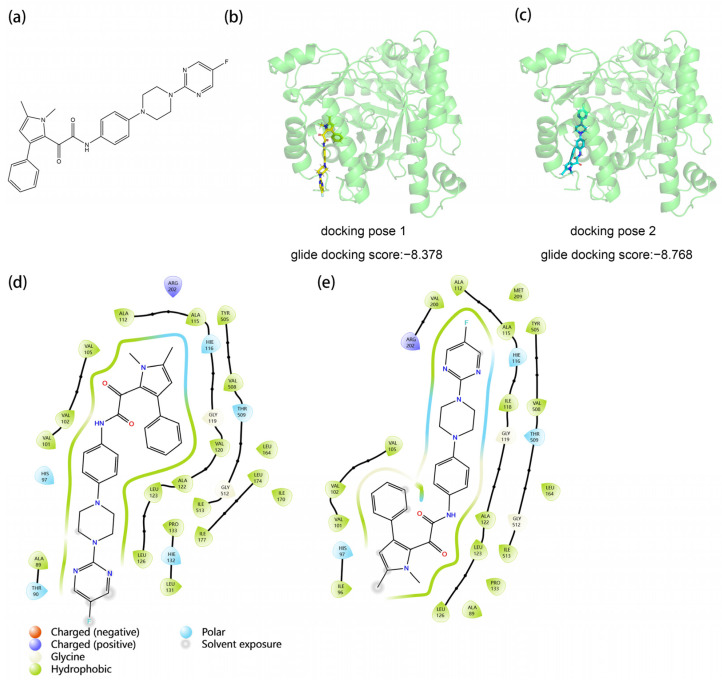
The structure of the Olorofim and the docking poses in the pocket of AfDHODH. (**a**) The structure of Olorofim; (**b**) pose 1 and (**c**) pose 2 of Olorofim docking positions; (**d**) 2D ligand interaction diagram of pose 1; (**e**) 2D ligand interaction diagram of pose 2.

The structure of Olorofim and two classes of AfDHODH-small molecule inhibitor (Olorofim) complex structures obtained via molecular docking are illustrated in Figure 1. These structures emerged from multiple docking trials exploring varying docking centers and box radii, with clustering analysis yielding two representative structural classes. Structural alignment revealed that Olorofim adopts an elongated T-shaped conformation, which drives the distinct docking outcomes. In pose 1 (Glide docking score = −8.378), the “T-head” is docked into the binding pocket, while in pose 2 (Glide docking score = −8.768), the slender “T-tail” occupies the pocket. In Figure 1d,e, it is not difficult to observe that both poses mainly rely on hydrophobic interactions to bind into the pocket, with the main difference being which end of the tail and head faces the solvent side: in pose 1, the tail faces the solvent, while in pose 2, the head faces the solvent, and both lack special interactions. Fifteen patent-derived small molecules (Lig1–15) were similarly docked, retaining their top 4 or 5 poses (see Figures S2 and S3). In the absence of a reference crystal structure for the complex, molecular docking results—though favoring pose 2 over pose 1 in scoring—remain static and fail to reflect dynamic binding processes under physiological conditions. To validate the rationality of the predicted protein–ligand interactions and identify the most biologically plausible binding mode, dynamic conformational analysis is essential. Consequently, molecular dynamics (MD) simulations were conducted to assess complex binding stability and structural flexibility.

### 2.2. Stability Analysis

**Figure 2:**
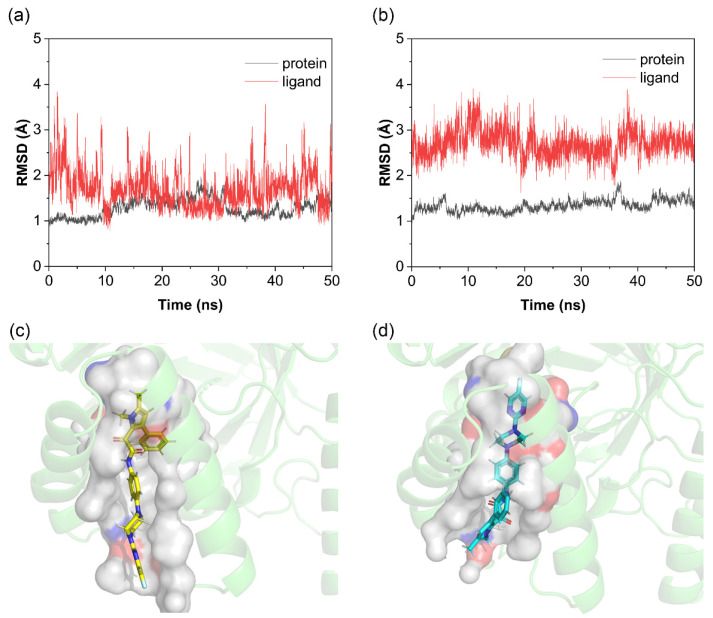
RMSD of Olorofim docking poses and binding pocket analysis. (**a**) RMSD of pose 1; (**b**) RMSD of pose 2; (**c**) binding pocket of pose 1; (**d**) binding pocket of pose 2.

As shown in Figure 2a,b, the 50 ns molecular dynamics simulations evaluating complex conformational stability revealed the following: For the protein backbone, both pose 1 and pose 2 exhibited minimal overall fluctuations (~1.5 Å). For the small molecule, the RMSD of pose 1 remained predominantly below 2 Å but with significant fluctuations, whereas pose 2 showed higher RMSD values (~2.7 Å) with reduced fluctuations. In this system, RMSD variations arose from the flexibility of the T-shaped tail of the Olorofim molecule. As shown in Figure 2c,d, in pose 1, the tail resides in the solvent-exposed region outside the binding pocket, allowing free movement, while the rigid T-shaped head (with aromatic rings) stably fits within the pocket. In contrast, pose 2 positions the rigid T-head outside the pocket and the flexible tail deep inside, restricting tail mobility. This results in reduced RMSD fluctuations for pose 2 but poorer shape complementarity and weaker binding, leading to higher overall RMSD values. Notably, in some simulations, pose 2 dissociated from its initial position and relocated to the FMN cofactor site.

Based on these observations, pose 2 was excluded from subsequent analyses, with pose 1 retained as the reference structure. For Lig1–15, in Figure S4, given the critical role of MD simulations in assessing receptor–ligand dynamic stability and the precedent set by Olorofim pose selection, this study diverged from the conventional practice of selecting top-scoring docking poses. Due to the absence of reference structures and validated binding modes, to mitigate energy calculation biases from conformational sampling limitations, the top 4–5 docking poses were subjected to MD simulations. Trajectories with minimal RMSD fluctuations were retained for subsequent free energy calculations, reducing false-positive risks from insufficient conformational sampling.

### 2.3. Correlation Between Experimental and Calculated Binding Energies

**Figure 3:**
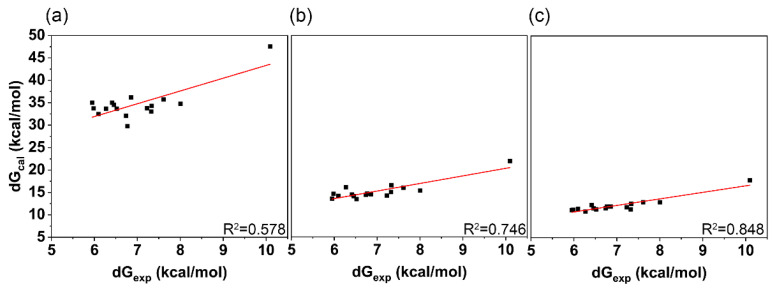
Correlation of different calculated methods with experimental values, with black dots representing actual data points and red lines representing fitted curves from linear regression. (**a**) MMGBSA; (**b**) ASGB; (**c**) ASGBIE.

In the last 20 ns of the MD simulation trajectory obtained in the previous step, we calculated the binding free energy of the ligand small molecule to the receptor using three methods (MMGBSA/ASGB/ASGBIE) to measure the tightness of the binding between the ligand and protein. As shown in Figure 3, We compared these calculated values with the experimental IC_50_-derived ∆G_exp_ to analyze their correlation. Due to differences in signs, only the absolute values of the binding free energies calculated by all methods were compared; the larger the absolute value, the tighter the binding. In terms of correlation, the MMGBSA method had the lowest Pearson correlation coefficient (R^2^ = 0.578, Figure 3a). After introducing alanine scanning (ASGB), the correlation significantly improved (R^2^ = 0.746, Figure 3b), as alanine scanning can largely offset systematic errors, leading to more stable and reliable results. With the interaction entropy (IE) correction, the ASGBIE method showed the highest Pearson correlation coefficient with the experimental binding free energy derived from IC_50_ (R^2^ = 0.848, Figure 3c). In terms of absolute values, the ASGBIE method was also closest to the experimental values, ranging between 10 and 18 kCal/mol, while MMGBSA was farthest from the experimental values, with calculated values between 30 and 40 kcal/mol. Considering both correlation with experimental values and absolute values, the ASGBIE method performed best among these methods and was closest to the actual situation. Therefore, in subsequent virtual screening, we will only use the ASGBIE method to calculate binding free energies.

### 2.4. Binding Pocket Hotspot Residues and Conservation Analysis

**Figure 4:**
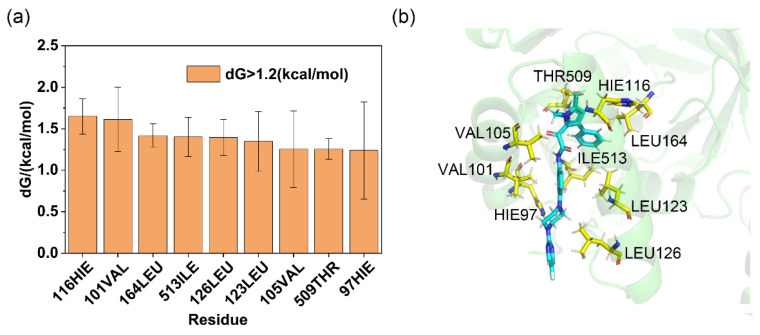
Residue binding free energy contributions, with distribution frequency. (**a**) Contribution of residues around the binding pocket to the binding free energy of Olorofim (residues contributing less than 1.0 kCal/mol were not labeled); (**b**) 3D structure of Olorofim and residues from binding site within 5 Å, the blue color denotes the ligand molecule, and the yellow amino acid residues are the hot spots.

For the complexes, the residue decomposition analysis of binding free energy calculated using the ASGBIE method is shown in Figure 4a. Residues with binding free energy contributions > 1.2 kcal/mol were defined as hotspot residues, while those with contributions < 0.8 kcal/mol were classified as cold spot residues. This study focused on key residues in the binding mode of AfDHODH with Olorofim; additional results are provided in Figure S4 and S5. The results indicate that for the AfDHODH-Olorofim complex, critical hotspot residues include 116HIE, 101VAL, 164LEU, 513ILE, 126LEU, 123LEU, 105VAL, 509THR, and 97HIE. Among these, residues 116HIE and 164LEU recurrently emerged as hotspots in the binding modes of Lig1–15 (frequencies: 86.7% and 80%, respectively). The hydrophobic side chains of these residues predominantly contributed to ligand binding via van der Waals interactions (2.2578 ± 0.2275 kcal/mol and 1.8229 ± 0.1005 kcal/mol, respectively), constituting the major energetic drivers (Table S1). Residues 116HIE and 164LEU also appeared as shared hotspots in subsequent residue decomposition analyses of binding free energy for virtual screening hits, suggesting their pivotal role in inhibitor binding. In contrast, hydrogen bond interactions, though typically crucial for ligand recognition, were unstable in the complex. The hydrogen bond formed by 115ALA was observed in ~50% of simulations, while that involving 202ARG occurred in <1% of cases. Their low energetic contributions and non-hotspot classification align with this transient behavior.

As illustrated in Figure 4b, three-dimensional topological analysis of the AfDHODH binding pocket reveals that its catalytic domain is primarily formed by two antiparallel α-helices, creating a highly narrow hydrophobic channel. This unique geometric constraint enables the elongated T-shaped Olorofim to tightly embed into the channel via hydrophobic interactions, likely explaining its potent competitive inhibitory activity. As the rate-limiting enzyme in the de novo pyrimidine biosynthesis pathway, AfDHODH’s functional non-redundancy and species-specific topological features of its binding pocket make it an ideal target for developing antifungal drugs against invasive fungal infections. The hotspot residue distribution and channel geometric constraints uncovered in this study provide critical theoretical foundations for the structure-based design of AfDHODH inhibitors.

### 2.5. Analysis of Virtual Screening

Given the clinical severity of invasive *Aspergillus fumigatus* infections and the limitations of current therapies, leveraging virtual screening to identify novel lead compounds from ultra-large chemical libraries represents a critical strategy to overcome therapeutic bottlenecks. Based on the previously established three-dimensional structure of the AfDHODH-Olorofim complex and the hotspot residue map, this study systematically screened the ZINC20 and ChEMBL databases. Both receptor-based and ligand-based virtual screening methods were employed to comprehensively explore potential lead compounds. Screening results were retained only for molecules exhibiting stable trajectories in MD simulations and ASGBIE-calculated binding free energy (ΔG_cal_) below −16 kcal/mol (except for the 2D Morgan fingerprint method, where the threshold was set at −14 kcal/mol). The retained hits are listed in Table S2. A total of 31 small molecules were identified, of which 23 were obtained through receptor-based virtual screening. Ligand-based virtual screening methods yielded only 8 hits, which generally exhibited weaker calculated binding free energies. These findings suggest that the homology model of AfDHODH, constructed under high-confidence parameters, is structurally reasonable, and the binding pocket/hotspot residue analyses are relatively accurate. Ligand-based approaches may inadequately account for pocket compatibility, leading to suboptimal outcomes. Docking scores and binding free energies for hits from each method are detailed in Tables S3–S5. Most retained molecules feature linear or T-shaped hydrophobic backbones with elongated carbon chains, matching the pocket geometry to facilitate deep penetration and robust hydrophobic interactions with pocket residues. Subsequent in vitro enzymatic activity assays were conducted to further validate these hypotheses.

### 2.6. Analysis of In Vitro Activity Results

**Figure 5:**
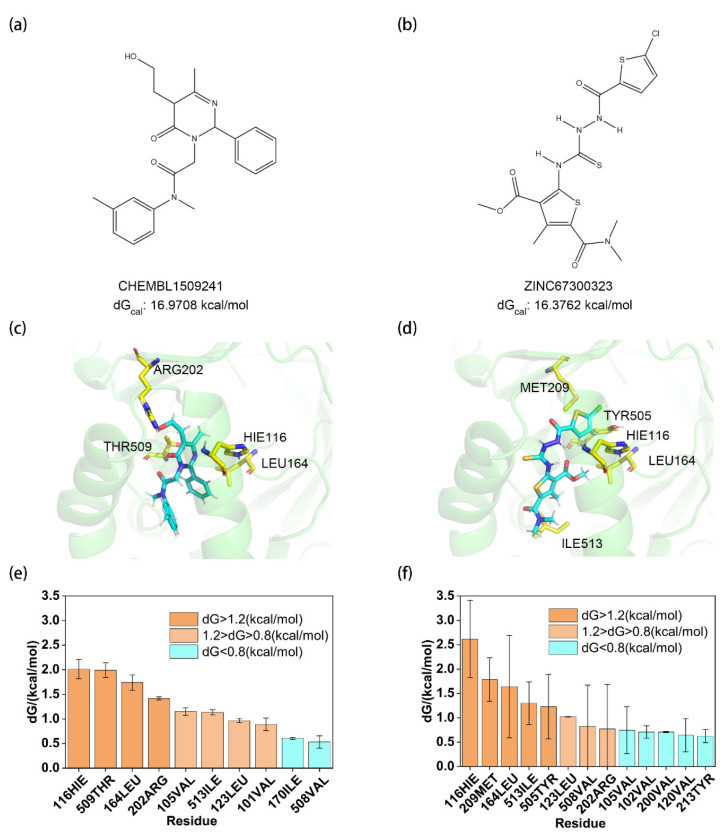
Analysis of the binding mode of the hit compounds. (**a**) The chemical structural formula and the calculated binding energy of CHEMBL1509241; (**b**) the chemical structural formula and the calculated binding energy of ZINC67300323; (**c**) the 3D structure of the hotspot residues bound to CHEMBL1509241, the blue color denotes the ligand molecule, and the yellow amino acid residues are the hot spots. (**d**) The binding free energy decomposition of CHEMBL1509241; (**e**) the 3D structure of the hotspot residues bound to ZINC67300323. (**f**) The binding free energy decomposition of ZINC67300323 (residues contributing less than 0.5 kcal/mol were not labeled).

As shown in Figure 5, this study evaluated the in vitro enzymatic activity of 13 commercially available small molecules from virtual screening results (CHEMBL1514025, ZINC000009641177, ZINC000067300707, ZINC000009640749, ZINC000002691260, ZINC000067301214, ZINC000067301018, ZINC000067300621, CHEMBL1509241, ZINC67300323, CHEMBL468176, ZINC20897666, ZINC000329326969) using the DCIP colorimetric assay. Inhibition percentages and IC_50_ values were calculated by monitoring DCIP absorbance changes at 600 nm. Notably, due to inherent limitations of the DCIP method, molecules capable of oxidizing DCIP may interfere with the accurate determination of initial reaction rates and inhibition percentages, a challenge that is difficult to eliminate in current screening workflows. All results are summarized in Table S22. The screening revealed inhibitory activity for compounds CHEMBL1509241 and ZINC67300323, both derived from receptor-based virtual screening, with IC_50_ values of 16.86 μM and 57.33 μM, respectively. These micromolar-level IC_50_ values suggest potential for structural optimization.

For molecular features, both compounds exhibit distinct scaffold architectures compared to Olorofim, with shorter molecular lengths. Their calculated binding free energies (16.9708 kcal/mol for CHEMBL1509241 and 16.3762 kcal/mol for ZINC67300323) align with their reduced molecular sizes. For binding mode, both molecules share similarities in binding pocket positioning, with rigid, bulky moieties anchored deep within the pocket. Hydrophobic interactions with residues 116HIE and 164LEU dominate stability, classifying these as hotspot residues. Residue 116HIE contributes the highest binding energy (>2.0 kcal/mol for both), followed by 164LEU (>1.5 kcal/mol). These residues also frequently appear as hotspots in Olorofim and Lig1–15 complexes due to favorable hydrophobic and shape complementarity. Residue 123LEU acts as a shared warm-spot residue to help them bind with a protein. Unique hotspots differentiate the two compounds: CHEMBL1509241 interacts with 202ARG and 509THR, while ZINC67300323 engages 209MET, 505TYR, and 513ILE. Structural differences drive these variations: ZINC67300323’s elongated backbone positions 513ILE (near the pocket exterior) as a unique hotspot, whereas CHEMBL1509241’s lateral extension elevates 202ARG (a warm-spot in ZINC67300323) to a hotspot. Proximity to the α-helix region further stabilizes 209MET in ZINC67300323.

For energetic correlation with activity, compared to Olorofim, the active compounds exhibit fewer hotspot residues (4 vs. 9), reducing total binding free energy by ~2 kcal/mol. This energy difference correlates well with the observed three-order-of-magnitude decrease in enzymatic activity in vitro, validating the structural and energetic rationale for their inhibitory potency.

It is critical to emphasize that while virtual screening did not achieve full success, computational practice demonstrates that the rationality of the initial structural model significantly impacts screening outcomes. The current results indicate that, in the AfDHODH system, homology modeling methods—exemplified by AlphaFold2—can yield reasonably accurate predictions for targets lacking crystal structures, provided sufficient homologous sequences and high-confidence binding pocket predictions are available. By leveraging a validated initial protein model and molecular docking, we can generate plausible predictions for protein–ligand binding modes. Subsequently, based on molecular dynamics (MD), dynamic analysis could effectively simulate physiological binding-dissociation processes, enabling the selection of biologically relevant complex conformations. These methodologies offer a rational pathway for virtual screening in protein–ligand systems with missing structural data, addressing challenges in structural and binding site information gaps.

Another key issue is that ligand-based virtual screening struggles to yield effective hits unless the pharmacophore–receptor binding profile of the lead molecule is validated. During drug development, extensive structural modifications are inevitable as candidates progress toward clinical use, often resulting in screened molecules with low similarity to the parent compound—or even lacking its core scaffold. Such molecules frequently fail to demonstrate promising activity in experimental validation.

Finally, hit compounds identified through virtual screening require structural optimization, including but not limited to: solubility enhancement (e.g., introducing polar groups); targeted pharmacophore modification (optimizing binding specificity via bioisosteric principles); steric hindrance modulation (adjusting molecular conformation to improve target fit), etc. These strategies aim to significantly enhance inhibitory efficacy by strengthening hydrogen-bond networks, optimizing hydrophobic complementarity, and aligning with target-specific topological features.

## 3. Methods

**Figure 6:**
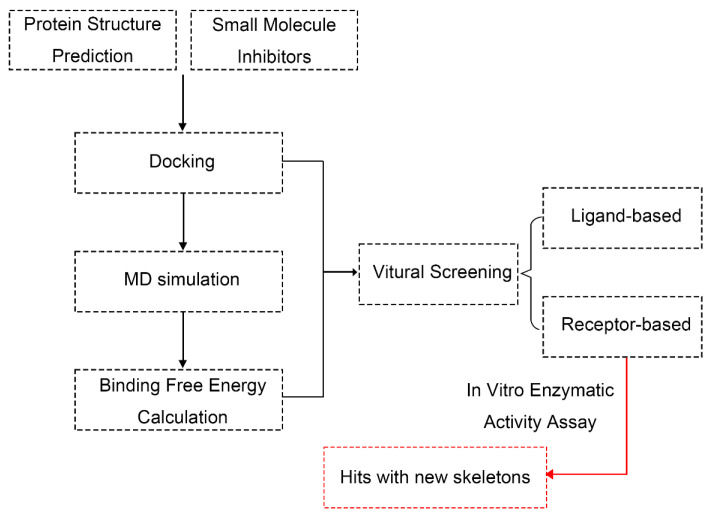
Computational workflow for AfDHODH-centric antifungal drug discovery: virtual screening, binding free energy analysis, and hits.

The overall procedure in the current study can be summarized in the flow chart in Figure 6. Specific descriptions of each step are discussed in the following sections.

### 3.1. Homology Modeling of Aspergillus fumigatus DHODH

Three-dimensional structure prediction of AfDHODH is based on the amino acid sequence of AfDHODH (UniProt accession number: Q4X169; sequence length: 531 AA) retrieved from the UniProt database. The UniProt database is a comprehensive resource for protein sequences and functional annotations, maintained by the European Bioinformatics Institute (EMBL-EBI), the Swiss Institute of Bioinformatics (SIB), and the Protein Information Resource (PIR). The latest version of the database was updated on 23 April 2025. The author’s access time was May 2023, and users can access the most recent data and related information through its official website (https://www.uniprot.org/, accessed on 10 June 2025). AlphaFold2 [17] was employed to predict its structure. As shown in Figure S1, among the five candidate models generated by the algorithm, the structural reliability was evaluated using the predicted Local Distance Difference Test (pLDDT). Given the minimal score differences between the five generated conformations and the well-overlapping binding pockets of the protein, Model 1—which exhibited slightly better scoring at the two α-helices (residues 96–128) near the entrance of the binding pocket—was selected for subsequent studies. To enhance model accuracy and reduce system equilibration time, low-confidence loop regions (residues 1–88 and 435–474) were removed in PyMOL (Schrödinger, LLC, New York, NY, USA). The optimized structure was then used for molecular docking and subsequent molecular dynamics simulations.

### 3.2. Virtual Screening

Target Preparation and Screening Strategy: For AfDHODH, the most stable Olorofim–AfDHODH complex obtained from molecular dynamics simulations was selected. A docking grid with a 25 Å radius was defined, centered on the ligand-binding site. The screened small molecule databases included ZINC20 (commercially available compounds, prepared for Deep Docking-accelerated virtual screening, provided by the Irwin and Shoichet Laboratories, University of California, San Francisco (UCSF), CA, USA, approximately 7.5 million compounds) and ChEMBL (public database, maintained by the European Molecular Biology Laboratory, Heidelberg, Germany, approximately 3 million compounds) [34,35].

Virtual Screening Workflow: All small molecules were processed using the LigPrep module (from 2022-2 Maestro, Schrödinger, LLC, New York, NY, USA) to adjust protonation states (pH = 7.4 ± 0.5), enumerate stereoisomers, and perform energy minimization, with force field parameters consistent with those used in docking calculations. The preprocessed molecules were then subjected to virtual screening using both receptor-based and ligand-based approaches.

Receptor-Based Virtual Screening: The receptor-based virtual screening was performed using the Virtual Screening Workflow module in Maestro. Specifically, QikProp was first applied to exclude small molecule ligands violating the Lipinski’s Rule. Three rounds of docking were then executed: (1) High-Throughput Virtual Screening (HTVS) docking was performed, retaining the top 10% of ligands by docking score; (2) Standard Precision (SP) docking was applied to the remaining ligands, with the top 10% selected; (3) Extra Precision (XP) docking was conducted for the final refinement. The resulting complexes were subjected to molecular dynamics simulations and binding free energy calculations to ensure structural stability and evaluate inhibitory potential by comparing their binding free energies with that of Olorofim. All results with absolute binding free energy values greater than 16 kCal/mol were retained.

Ligand-Based Virtual Screening:

The 3D Structure-Based Ligand Screening: For 3D structure-based ligand virtual screening, Maestro’s Shape Screening component was employed. Using the most stable conformation of Olorofim docked in the enzyme as a template (see Section 3.3 for details), a typed pharmacophore was generated and applied for virtual screening, with all other parameters set to default. Results were retained if their absolute binding free energy exceeded 16 kcal/mol, followed by the same computational and evaluation procedures as the receptor-based virtual screening.

The 2D Structure-Based Ligand Screening: For 2D structure-based ligand screening, RDKIT (RDKit is an open-source cheminformatics library developed by the OpenEye Scientific Software group, OpenEye, San Diego, CA, USA, version 2017.09.1) was first used to generate SMILES strings for the template molecule (Olorofim) and the screened molecules. Morgan fingerprints were generated with a radius of 2 (radius = 2) and a bit length of 2048 (nBits = 2048) to balance feature resolution and computational efficiency. Tanimoto coefficients were calculated to assess similarity between screened molecules and the target, retaining molecules with similarity scores above 0.25. These were subjected to the same computational and evaluation steps as the 3D-based method, with final retention of results showing absolute binding free energy values greater than 14 kcal/mol.

### 3.3. Molecular Docking

The structure of the small-molecule inhibitor Olorofim was retrieved from PubChem (https://pubchem.ncbi.nlm.nih.gov/, accessed on 10 June 2025, Compound CID: 91885568), while the remaining 15 small molecules were sourced from a 2009 patent by F2G Ltd. (Eccles, UK) [36]. Among the virtual screening results, the small molecule structures were derived from retained hits in the screened databases. The receptor-based virtual screening method retained 23 small molecules, while the ligand-based approach retained 8 small molecules (see Table S2). All ligands were docked using the Glide module in Schrödinger 2022-2 Maestro [37,38,39,40,41]. The workflow included the following:

Receptor Preparation: The modeled AfDHODH structure was preprocessed via Protein Preparation Wizard with the OPLS 2005 force field. Hydrogen atoms were added, protonation states were optimized at pH 7.4, and energy minimization was performed.

Grid Generation: A docking grid centered at coordinates (x, y, z) = (−12.57, −12.30, −0.23) with a 25 Å radius was generated using the Receptor Grid Generation module to encompass the binding pocket.

Ligand Preparation: Ligands, including Olorofim, were optimized using the LigPrep module with the OPLS 2005 force field at a pH of 7.4. Hydrogen addition, energy minimization, and isomer generation were performed.

Docking Execution: Flexible docking was conducted in Standard Precision (SP) mode. In the docking process of Olorofim, multiple results were clustered, and the two most typical docking poses were retained; for Lig1-15, the first four to five docking poses were retained. These results were used for further calculations and analysis.

### 3.4. Molecular Dynamics Simulations

Molecular dynamics (MD) simulations were employed to validate the stability of protein–small molecule ligand complexes, including Olorofim and the screened molecules from virtual screening (23 from receptor-based and 8 from ligand-based methods). Molecular dynamics (MD) simulations were used to verify the stability of the protein–small molecular ligand complex, including Olorofim and molecules from the virtual screen, which included two steps: first, to verify the binding pocket structure of the target, that is, to perform molecular dynamics simulations on the results of the docking of Olorofim and Lig1-15 for validation to verify whether the docking structure was reasonable; the second step is based on the results of the first step’s validation, and the same approach is taken in the virtual screening step to rule out unstable results in molecular dynamics simulations. The detailed procedures are as follows:

System Setup and Parameterization:

For the protein–ligand complex, hydrogen atoms and protonation states were optimized using the Proteins Plus server [42,43]. Ligand geometries were refined at the HF/6-31G* level with Gaussian16 Rev. C.01 [44] to derive RESP charges. The complex system was parameterized with the ff14SB force field for the protein [45] and the GAFF force field for the ligand [46]. The solvated system was embedded in a truncated octahedral TIP3P water box with a 12.0 Å buffer distance, followed by charge neutralization using chloride (Cl⁻) and sodium (Na⁺) ions in AmberTools18 with LEaP.

Energy Minimization and Equilibration:

The system underwent energy minimization using conjugate gradient and steepest descent algorithms to eliminate steric clashes between solute and solvent. Non-bonded interactions were truncated at 10 Å. Solvent molecules were first optimized with a 500 kcal/(mol·Å^2^) restraint on solute coordinates, followed by the unrestrained minimization of the entire system. Post-minimization, all atoms of the receptor–ligand complex were constrained with a 5 kcal/(mol·Å^2^) force constant. The system was heated from 0 K to 300 K over 300 ps, followed by a 25 ps NVT ensemble equilibration under the same restraints. A 500 ps NPT ensemble pre-equilibration without restraints preceded production runs. Finally, 50 ns production MD simulations were performed. Throughout minimization and equilibration, hydrogen bonds were constrained via the SHAKE algorithm, pressure was maintained at 1.0 atm using the Berendsen barostat, temperature was regulated by Langevin dynamics, and periodic boundary conditions were applied. The integration step was set to 2 fs. To enhance statistical significance and reduce random errors, three independent replicates were conducted. Trajectories were saved with only protein and ligand atoms retained, excluding solvent molecules.

### 3.5. Trajectory Analysis and Binding Free Energy Calculations

Trajectory Analysis: Using the CPPTRAJ module in AMBER18, trajectory analysis focused on Root Mean Square Deviation (RMSD). The RMSD of protein backbone Cα atoms and ligand heavy atoms was calculated relative to the initial structure across the entire 50 ns trajectory, with snapshots extracted every 10 frames. System stability was defined as RMSD fluctuations ≤2.0 Å during the final 20 ns.

Binding Free Energy Calculations: A total of 500 frames (1 frame per 100 ps) were extracted from equilibrated MD trajectories. MM/PB(GB)SA calculations were performed using the MMPBSA.py module in AMBER18, which computes the energy difference between the protein–ligand complex and unbound states while decomposing contributions from distinct interactions. The protocol followed our previous work on human DHODH [47] with binding free energy (∆G) calculated as:

(1) $$∆G_{bind}=∆E_{MM}+∆G_{solv}-T∆S$$

Here, $∆E_{MM}$ includes $∆E_{internal}\;$(bond, angle, and dihedral energies),$\;∆E_{elect}\;$(electrostatic energy), and $∆E_{vdwl}$ (van der Waals energy); $∆G_{solv}$ represents solvation free energy, comprising polar and nonpolar contributions. The polar term was calculated using the Generalized Born (GB) model, while the nonpolar term was estimated via the solvent-accessible surface area (SASA) method. Conformational entropy change (T∆S) is typically computed through normal mode analysis, but due to its high computational cost and poor correlation, we instead employed the interaction entropy (IE) method [32]. The interaction entropy is defined as:

(2) $$-T∆S=KTln\langle e^{\beta ∆E_{pl}^{int}}\rangle$$

In addition, alanine scanning serves as a critical computational approach. Alanine, the simplest amino acid residue, minimally perturbs a protein’s spatial structure or electrostatic effects. By mutating surface residues within 5 Å of the binding pocket to alanine, the resulting differences in binding free energy effectively reveal the functional contributions of these residues to ligand binding. The binding energy contribution of a specific residue is defined as the difference in binding free energy before and after mutation:

(3) $$∆∆G_{bind}^{x\to a}=∆G_{bind}^{a}-∆G_{bind}^{x}=∆∆G_{gas}^{x\to a}+∆∆G_{sol}^{x\to a}$$

Here, $∆∆G_{bind}^{x\to a}\;$ represents the contribution of residue x to the binding free energy, where $∆G_{bind}^{x}$ is the binding free energy between the wild-type protein and ligand. $∆G_{bind}^{a}$ is the binding free energy after mutating residue x to alanine. The total free energy is calculated as the sum of gas-phase and solvation contributions:

(4) $$∆∆G_{gas}^{x\to a}=∆G_{gas}^{a}-∆G_{gas}^{x}$$

(5) $$∆∆G_{sol}^{x\to a}=∆G_{sol}^{a}-∆G_{sol}^{x}$$

Here, $∆G_{gas}^{x}$ is gas-phase binding energy, $∆G_{sol}^{x}$ is solvation energy; similarly, they are calculated as differences between wild-type and alanine-mutated systems. Thus, the total binding free energy is derived by summing contributions from all residues near the binding pocket through alanine scanning. $∆G_{gas}^{x}$ and alanine mutant $∆G_{gas}^{a}$ were computed using the interaction entropy (IE) method:

(6) $$∆G_{gas}^{x}=\langle E_{pl}^{x}\rangle -T∆S_{pl}^{x}=\langle E_{pl}^{x}\rangle +KTln\langle e^{\beta ∆E_{pl}^{x}}\rangle$$

(7) $$∆G_{gas}^{a}=\langle E_{pl}^{a}\rangle -T∆S_{pl}^{a}=\langle E_{pl}^{a}\rangle +KTln\langle e^{\beta ∆E_{pl}^{a}}\rangle$$

In Formulas (6) and (7), The interaction energies between the ligand and residues x and a are denoted as $E_{pl}^{x}$ and $E_{pl}^{a}$, respectively:

(8) $$\langle e^{\beta ∆E_{pl}^{x}}\rangle =\frac{1}{N}\sum_{i=1}^{N}e^{\beta ∆E_{pl}^{x}\left(t_{i}\right)}$$

N denotes the number of snapshots extracted from the molecular dynamics simulation trajectory. Therefore, in Formula (4), the gas-phase binding free energy formula for a specific residue is expressed as:

(9) $$∆∆G_{gas}^{x\to a}=∆∆E_{gas}^{x\to a}-T∆∆S_{gsa}^{x\to a}=\langle E_{pl}^{a}\rangle -\langle E_{pl}^{x}\rangle +\left[ln\langle e^{\beta ∆E_{pl}^{a}}\rangle -ln\langle e^{\beta ∆E_{pl}^{x}}\rangle \right]$$

The polar component of solvation energy was calculated using the “IGB = 2” model, where the dielectric constants for nonpolar, polar, and charged residues were set to 1, 3, and 5, respectively. The total binding free energy was computed as the sum of contributions from each residue within the 5 Å binding pocket [33]:

(10) $$∆G_{bind}=-\sum_{x}∆∆G_{bind}^{x\to a}$$

In subsequent discussions, the MM/GBSA method incorporating alanine scanning is abbreviated as ASGB, while the GBSA method with IE-based entropy correction is termed ASGBIE. For correlating calculated binding free energies with experimental values, we utilized IC_50_ obtained from patents and computed the Pearson correlation coefficient. IC_50_ is the half maximal inhibitory concentration, defined as the concentration of an inhibitor required to reduce a biochemical function (such as enzyme activity, cell proliferation, or receptor activation) by 50% in vitro. Lower values indicate the stronger inhibitory potency of the compound. In small-molecule drug design, IC_50_ is primarily used to evaluate in vitro inhibition of enzyme activity. Here, experimental binding free energy was derived from IC_50_ using the formula:

(11) $$∆G_{exp}=RT\cdot {lnK}_{d}\approx RT\cdot ln{IC}_{50}$$

with *R* = 8.314 J∙mol^−1^∙K^−1^ and temperature *T* in Kelvin. In this work, the temperature is set at 300 K (room temperature). In the subsequent calculation of binding free energy, to maintain consistency, the sign of $∆G_{exp}$ is unified with the calculated value. Specifically, the binding free energy calculated using MM/GBSA is not corrected, meaning $∆G_{exp}$ remains negative; whereas when calculated using the ASGBIE method, $∆G_{exp}$ is corrected by adding a negative sign, resulting in a positive value. In either case, a higher absolute value of $∆G_{exp}$ indicates better small molecule inhibition. In subsequent binding free energy calculations, the sign convention of $∆G_{cal}$ was aligned with $∆G_{exp}$ for consistency. Specifically, binding free energies calculated using MM/PB(GB)SA were corrected to positive values (i.e., $∆G_{cal}$ is positive), whereas no correction was applied to the ASGBIE method because $∆G_{cal}$ inherently yields positive values. In all cases, higher absolute values of $∆G_{exp}$ indicate stronger inhibitory effects of the small molecules.

### 3.6. In Vitro Activity Assay

#### 3.6.1. Experimental Methods

The catalytic activity of dihydroorotate dehydrogenase (DHODH) was determined using the DCIP colorimetric assay (2,6-dichloroindophenol reduction method), based on the redox process in the enzymatic electron transport chain. The principle is as follows:

Under DHODH catalysis, two protons and electrons from the substrate L-dihydroorate (DHO) are transferred to the flavin mononucleotide (FMN) cofactor, generating reduced FMNH_2_. FMNH_2_ subsequently donates electrons to free coenzyme Q (CoQ, ubiquinone), which ultimately transfers electrons to the chromogenic substrate DCIP, reducing it. Oxidized DCIP exhibits maximal absorption at 600 nm, while reduced DCIP shows no absorption at this wavelength. Thus, the oxidation rate of DHO is quantified by monitoring the decrease in absorbance at 600 nm, from which the initial enzymatic reaction velocity (V_0_) is calculated. Upon inhibitor addition, the initial velocity (V_i_) decreases. Inhibition percentage is calculated using the formula $\left(1-V_{i}/V_{0}\right)$, and IC_50_ values are derived.

#### 3.6.2. Experimental Procedures

Following the protocol from F2G Ltd. [14], the assay buffer contained 10% wt/vol glycerol (Aladdin, Riverside, CA, USA, G116214-100 mL), 0.05 M of Tris-HCl (Rhawn, Shanghai, China, R198482-100 mL, pH 8.0), 0.15 M of KCl (Guogen Chemicals, Shanghai, China, 10016318), 0.1% wt/vol Triton X-100 (Sigma, St. Louis, MO, USA, X100-5 mL), supplemented with 50 μM of CoQ2 (Sigma, C8081-2 mg) and 100 μM of DCIP (Aladdin, D196076-200 mg). AfDHODH protein (residues 89–531 with an N-terminal His-tag, custom-synthesized by ACROBiosystems, Newark, DE, USA) was diluted to 1.736 μM in assay buffer. Small-molecule inhibitors, dissolved in ultrapure water or DMSO, were mixed with the enzyme solution and incubated at room temperature for 20 min to ensure inhibitor–enzyme binding. The reaction was initiated by adding L-DHO (Solarbio, Beijing, China, ID5640-100 mg, final concentration: 1 mM), and absorbance at 600 nm was monitored every 2 min for 20 min using a UV spectrophotometer (SHIMADZU, Kyoto, Japan, UV-2600). Control groups (without inhibitors) were assayed in parallel. Enzyme activity was calculated from the linear-phase absorbance change rate. Inhibition percentage and IC_50_ were determined using Formula (12):

(12) $$Inhibition\;\left(\%\right)=\left(1-\frac{V_{i}}{V_{0}}\right)\times 100\%$$

where V_0_ is the initial reaction rate of the control (no inhibitor) and V_i_ is the rate with the inhibitor. In actual measurements, since the path length and molar extinction coefficient are fixed values, the reaction rate depends only on time and the change in absorbance. In the actual calculation of reaction rates, the absorbance measured at 600 nm at the end of the reaction (24 h or longer) is considered to be zero concentration, and the absorbance at the beginning of the reaction (time = 0) is considered to be 100% concentration. By subtracting the absorbance at the end of the reaction, the initial concentration of the substrate can be calculated, taking into account the zero-order reaction characteristics of AfDHODH during the catalytic L-DHO reaction. In the early stage of the reaction, due to the excess substrate and minimal product formation, the reaction initially exhibits zero-order behavior, meaning that the reaction rate remains constant over a certain period (typically within 30 min in this study). Therefore, the absorbance data obtained can be converted to concentration and normalized to the concentration–time curve, allowing for the calculation of the initial reaction rate $V_{0}$. However, when inhibited by an inhibitor, AfDHODH shows a certain degree of reduction in the initial reaction rate $V_{i}$, and the inhibition rate and IC_50_ can be calculated by determining the ratio of the initial reaction rates.

## 4. Conclusions

The current study addressed the challenge of the absence of a crystal structure for the *Aspergillus fumigatus* DHODH (AfDHODH) target by integrating computer-aided drug design (CADD) and experimental validation, establishing a comprehensive workflow from modeling to screening. A three-dimensional AfDHODH structure was first obtained and refined using homology modeling. Receptor–ligand complex binding modes were then constructed via molecular docking, with model reliability validated through molecular dynamics (MD) simulations and ASGBIE-based binding free energy calculations. By decomposing binding free energy contributions to residues within 5 Å of the binding site, hotspot residue distributions were identified, and binding modes were analyzed. Building on this framework, receptor- and ligand-based virtual screenings were conducted, and 13 candidate molecules were selected for in vitro enzymatic activity validation.

By experimental validation, two inhibitor molecules—CHEMBL1509241 and ZINC67300323—exhibiting chemical scaffolds distinct from Olorofim demonstrated micromolar-level inhibitory potency. Residue decomposition analysis revealed shared hotspot residues 116HIE and 164LEU, which stabilize inhibitor binding through hydrophobic interactions. This discovery not only confirms the efficacy of the virtual screening strategy but also breaks the structural limitations of existing inhibitors, providing a new starting point for antifungal drug optimization and design. Notably, the shared and unique hotspot residue patterns of these hits suggest potential molecular mechanisms of action, offering new directions for subsequent mechanistic studies.

For AfDHODH, this study established a robust computational pipeline—spanning homology modeling, MD simulations, trajectory analysis, binding free energy calculations, hotspot residue profiling, and virtual screening—coupled with experimental validation. This workflow enables drug discovery for structurally uncharacterized targets with sufficient homology information. While low experimental hit rates remain a challenge, future advances in high-resolution structural elucidation and structure–activity relationship studies will gradually resolve these limitations, offering innovative solutions to the escalating threat of antifungal resistance.

## Data Availability

The raw computational files and scripts used in this study are openly available in Zenodo at https://doi.org/10.5281/zenodo.15272616, under the Creative Commons Attribution 4.0 International (CC BY 4.0) license.

## Supplementary Materials

The following supporting information can be downloaded at: https://www.mdpi.com/article/10.3390/molecules30122607/s1. Figure S1 shows the AlphaFold2 modeling results of AfDHODH. Figures S2–S5 and Table S1 present the molecular docking, MD simulations, and binding free energy calculation results of lig1–15. Tables S2–S5 display the computational results from virtual screening. Tables S6–S21 contain the residue decomposition binding free energy calculation results between AfDHODH and small molecule ligands (Olorofim and lig1–15). Table S22 summarizes the in vitro enzymatic activity assay results.

## References

[1] Lamoth F., Glampedakis E., Boillat-Blanco N., Oddo M., Pagani J.-L. (2020). Incidence of Invasive Pulmonary Aspergillosis among Critically Ill COVID-19 Patients. Clin. Microbiol. Infect..

[2] Schauwvlieghe A.F.A.D., de Jonge N., van Dijk K., Verweij P.E., Brüggemann R.J., Biemond B.J., Bart A., von dem Borne P.A., Verbon A., van der Beek M.T. (2018). The Diagnosis and Treatment of Invasive Aspergillosis in Dutch Haematology Units Facing a Rapidly Increasing Prevalence of Azole-Resistance. A Nationwide Survey and Rationale for the DB-MSG 002 Study Protocol. Mycoses.

[3] Dupont D., Menotti J., Turc J., Miossec C., Wallet F., Richard J.-C., Argaud L., Paulus S., Wallon M., Ader F. (2021). Pulmonary Aspergillosis in Critically Ill Patients with Coronavirus Disease 2019 (COVID-19). Med. Mycol..

[4] Pasko M.T., Piscitelli S.C., Van Slooten A.D. (1990). Fluconazole: A New Triazole Antifungal Agent. Dicp.

[5] De Beule K. (1996). Itraconazole: Pharmacology, Clinical Experience and Future Development. Int. J. Antimicrob. Agents.

[6] Letscher-Bru V., Herbrecht R. (2003). Caspofungin: The First Representative of a New Antifungal Class. J. Antimicrob. Chemother..

[7] Lemke A., Kiderlen A.F., Kayser O. (2005). Amphotericin B. Appl. Microbiol. Biotechnol..

[8] Waldorf A.R., Polak A. (1983). Mechanisms of Action of 5-Fluorocytosine. Antimicrob. Agents Chemother..

[9] Van Daele R., Spriet I., Wauters J., Maertens J., Mercier T., Van Hecke S., Brüggemann R. (2019). Antifungal Drugs: What Brings the Future?. Med. Mycol..

[10] Sanglard D. (2016). Emerging Threats in Antifungal-Resistant Fungal Pathogens. Front. Med..

[11] Dagenais T.R.T., Keller N.P. (2009). Pathogenesis of Aspergillus Fumigatus in Invasive Aspergillosis. Clin. Microbiol. Rev..

[12] Pinder C., Lebedinec R., Levine T.P., Birch M., Oliver J.D. (2023). Characterisation of Putative Class 1A DHODH-like Proteins from Mucorales and Dematiaceous Mould Species. PLoS ONE.

[13] Zhu J., Thompson C.B. (2019). Metabolic Regulation of Cell Growth and Proliferation. Nat. Rev. Mol. Cell Biol..

[14] Oliver J.D., Sibley G.E.M., Beckmann N., Dobb K.S., Slater M.J., McEntee L., du Pré S., Livermore J., Bromley M.J., Wiederhold N.P. (2016). F901318 Represents a Novel Class of Antifungal Drug That Inhibits Dihydroorotate Dehydrogenase. Proc. Natl. Acad. Sci. USA.

[15] Vanbiervliet Y., Van Nieuwenhuyse T., Aerts R., Lagrou K., Spriet I., Maertens J. (2024). Correction: Review of the Novel Antifungal Drug Olorofim (F901318). BMC Infect. Dis..

[16] Wiederhold N.P. (2020). Review of the Novel Investigational Antifungal Olorofim. J. Fungi.

[17] Jumper J., Evans R., Pritzel A., Green T., Figurnov M., Ronneberger O., Tunyasuvunakool K., Bates R., Žídek A., Potapenko A. (2021). Highly Accurate Protein Structure Prediction with AlphaFold. Nature.

[18] Sabe V.T., Ntombela T., Jhamba L.A., Maguire G.E.M., Govender T., Naicker T., Kruger H.G. (2021). Current Trends in Computer Aided Drug Design and a Highlight of Drugs Discovered via Computational Techniques: A Review. Eur. J. Med. Chem..

[19] Karplus M., Petsko G.A. (1990). Molecular Dynamics Simulations in Biology. Nature.

[20] Frenkel D., Smit B., Tobochnik J., McKay S.R., Christian W. (1997). Understanding Molecular Simulation. Comput. Phys..

[21] Case D.A., Ben-Shalom I.Y., Brozell S.R., Cerutti D.S., Cheatham T.E., Cruzeiro V.W.D., Darden T.A., Duke R.E., Ghoreishi D., Gilson M.K. (2018). AMBER 2018.

[22] van der Kamp M.W., Shaw K.E., Woods C.J., Mulholland A.J. (2008). Biomolecular Simulation and Modelling: Status, Progress and Prospects. J. R. Soc. Interface.

[23] Woodley S.M., Battle P.D., Gale J.D., Richard ACatlow C. (1999). The Prediction of Inorganic Crystal Structures Using a Genetic Algorithm and Energy Minimisation. Phys. Chem. Chem. Phys..

[24] Bash P.A., Field M.J., Karplus M. (1987). Free Energy Perturbation Method for Chemical Reactions in the Condensed Phase: A Dynamic Approach Based on a Combined Quantum and Molecular Mechanics Potential. J. Am. Chem. Soc..

[25] Rao S.N., Singh U.C., Bash P.A., Kollman P.A. (1987). Free Energy Perturbation Calculations on Binding and Catalysis after Mutating Asn 155 in Subtilisin. Nature.

[26] Massova I., Kollman P.A. (1999). Computational Alanine Scanning to Probe Protein−Protein Interactions: A Novel Approach to Evaluate Binding Free Energies. J. Am. Chem. Soc..

[27] Massova I., Kollman P.A. (2000). Combined Molecular Mechanical and Continuum Solvent Approach (MM-PBSA/GBSA) to Predict Ligand Binding. Perspect. Drug Discov. Design.

[28] Beveridge D. (1989). Free Energy via Molecular Simulation: Applications to Chemical and Biomolecular Systems. Annu. Rev. Biophys. Biomol. Struct..

[29] Zacharias M., Straatsma T.P., McCammon J.A. (1994). Separation-Shifted Scaling, a New Scaling Method for Lennard-Jones Interactions in Thermodynamic Integration. J. Chem. Phys..

[30] Ramos R.M., Moreira I.S. (2013). Computational Alanine Scanning Mutagenesis-an Improved Methodological Approach for Protein-DNA Complexes. J. Chem. Theory Comput..

[31] Sun H., Li Y., Shen M., Tian S., Xu L., Pan P., Guan Y., Hou T. (2014). Assessing the Performance of MM/PBSA and MM/GBSA Methods. 5. Improved Docking Performance Using High Solute Dielectric Constant MM/GBSA and MM/PBSA Rescoring. Phys. Chem. Chem. Phys..

[32] Duan L., Liu X., Zhang J.Z.H. (2016). Interaction Entropy: A New Paradigm for Highly Efficient and Reliable Computation of Protein-Ligand Binding Free Energy. J. Am. Chem. Soc..

[33] Cong Y., Li M., Feng G., Li Y., Wang X., Duan L. (2017). Trypsin-Ligand Binding Affinities Calculated Using an Effective Interaction Entropy Method under Polarized Force Field. Sci. Rep..

[34] Zdrazil B., Felix E., Hunter F., Emma J., Manners J., Blackshaw S., Corbett M., De Veij H., Ioannidis D.M., Lopez J.F. (2015). ChEMBL Web Services: Streamlining Access to Drug Discovery Data and Utilities. Nucleic Acids Res..

[35] Irwin J.J., Tang K.G., Young J., Dandarchuluun C., Wong B.R., Khurelbaatar M., Moroz Y.S., Mayfield J., Sayle R.A. (2020). ZINC20—A Free Ultralarge-Scale Chemical Database for Ligand Discovery. J. Chem. Inf. Model..

[36] Oliver J., David T.J., Bromley M., John S.G.E., Morris B. (2009). Dihydroorotate Dehydrogenase as Antifungal Drug Target and Quinazolinone-Based Inhibitors Thereof. U.S. Patent.

[37] (2025). Schrödinger Release 2025-2.

[38] Yang Y., Yao K., Repasky M.P., Leswing K., Abel R., Shoichet B.K., Jerome S.V. (2021). Efficient Exploration of Chemical Space with Docking and Deep Learning. J. Chem. Theory Comput..

[39] Friesner R.A., Murphy R.B., Repasky M.P., Frye L.L., Greenwood J.R., Halgren T.A., Sanschagrin P.C., Mainz D.T. (2006). Extra Precision Glide: Docking and Scoring Incorporating a Model of Hydrophobic Enclosure for Protein-Ligand Complexes. J. Med. Chem..

[40] Halgren T.A., Murphy R.B., Friesner R.A., Beard H.S., Frye L.L., Pollard W.T., Banks J.L. (2004). Glide: A New Approach for Rapid, Accurate Docking and Scoring. 2. Enrichment Factors in Database Screening. J. Med. Chem..

[41] Friesner R.A., Banks J.L., Murphy R.B., Halgren T.A., Klicic J.J., Mainz D.T., Repasky M.P., Knoll E.H., Shelley M., Perry J.K. (2004). Glide: A New Approach for Rapid, Accurate Docking and Scoring. 1. Method and Assessment of Docking Accuracy. J. Med. Chem..

[42] Lippert T., Rarey M. (2009). Fast Automated Placement of Polar Hydrogen Atoms in Protein-Ligand Complexes. J. Cheminform..

[43] Bietz S., Urbaczek S., Schulz B., Rarey M. (2014). Protoss: A Holistic Approach to Predict Tautomers and Protonation States in Protein-Ligand Complexes. J. Cheminform..

[44] Frisch M.J., Trucks G.W., Schlegel H.B., Scuseria G.E., Robb M.A., Cheeseman J.R., Scalmani G., Barone V., Petersson G.A., Nakatsuji H. (2016). Gaussian 16.

[45] Maier J.A., Martinez C., Kasavajhala K., Wickstrom L., Hauser K.E., Simmerling C. (2015). Ff14SB: Improving the Accuracy of Protein Side Chain and Backbone Parameters from ff99SB. J. Chem. Theory Comput..

[46] Wang J., Wolf R.M., Caldwell J.W., Kollman P.A., Case D.A. (2004). Development and Testing of a General Amber Force Field. J. Comput. Chem..

[47] Bian H., Cong Y. (2023). Identification of Mechanism of Action and Novel Compounds Targeting HsDHODH: Insights from Computational Analysis. Biol. Med. Chem..

