# Supplementary material for: Discovery of Novel Inhibitors of Aspergillus fumigatus DHODH via Virtual Screening, MD Simulation, and In Vitro Activity Assay
Source: Molecules. 2025 Jun 16;30(12):2607. doi: 10.3390/molecules30122607 (PMC12196321; doi:10.3390/molecules30122607)
Supplement: Supplementary file 1 [file molecules-30-02607-s001.zip › molecules-3671571-supplementary.pdf]

molecules

MDPI

Supporting Information

Discovery of Novel Inhibitors of *Aspergillus fumigatus* DHODH via Virtual Screening, MD Simulation, and in Vitro Activity Assay

Kaige Li [1], Wei Xia [2,3] and John Z. H. Zhang [1,2,3,4,5,*]

[1] Shanghai Engineering Research Center of Molecular Therapeutics and New Drug, Development, Shanghai Key Laboratory of Green Chemistry & Chemical Process, School of Chemistry and Molecular Engineering, East China Normal University, Shanghai 200062, China; 51254300105@stu.ecnu.edu.cn
[2] NYU-ECNU Center for Computational Chemistry and Shanghai Frontiers Science Center of AI and DL, New York University Shanghai, Shanghai 200126, China; wx2237@nyu.edu
[3] Department of Chemistry, New York University, New York, NY 10003, USA
[4] Faculty of Synthetic Biology, Shenzhen University of Advanced Technology, Shenzhen 518107, China
[5] State Key Laboratory of Quantitative Synthetic Biology, Shenzhen Institute of Synthetic Biology, Shenzhen Institute of Advanced Technology, Chinese Academy of Sciences, Shenzhen 518055, China
* Correspondence: john.zhang@nyu.edu

Academic Editor: Maxim L. Kuznetsov

Received: 14 May 2025
Revised: 10 June 2025
Accepted: 11 June 2025
Published: 16 June 2025

Citation: Li, K.; Xia, W.; Zhang, J.Z.H. Discovery of Novel Inhibitors of *Aspergillus fumigatus* DHODH via Virtual Screening, MD Simulation, and In Vitro Activity Assay. *Molecules* **2025**, *30*, 2607. https://doi.org/10.3390/10.3390/molecules30122607

Copyright: © 2025 by the authors. Submitted for possible open access publication under the terms and conditions of the Creative Commons Attribution (CC BY) license (https://creativecommons.org/licenses/by/4.0/).

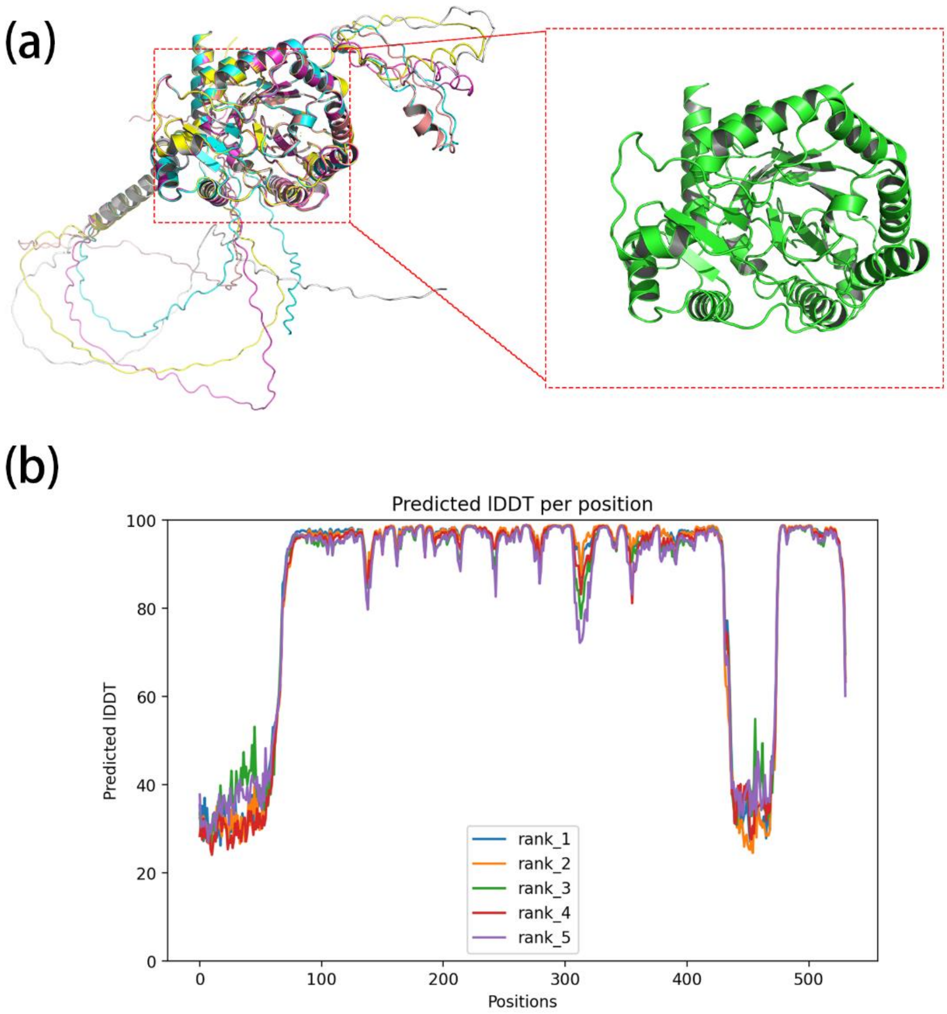

Figure S1. Model Selection and Optimization for AlphaFold2-Generated Structures. (a) the 5 model of AlphaFold2-Generated Structures, inside the red box are the optimized structures; (b) pLDDT of the 5 model of AlphaFold2-Generated Structures.

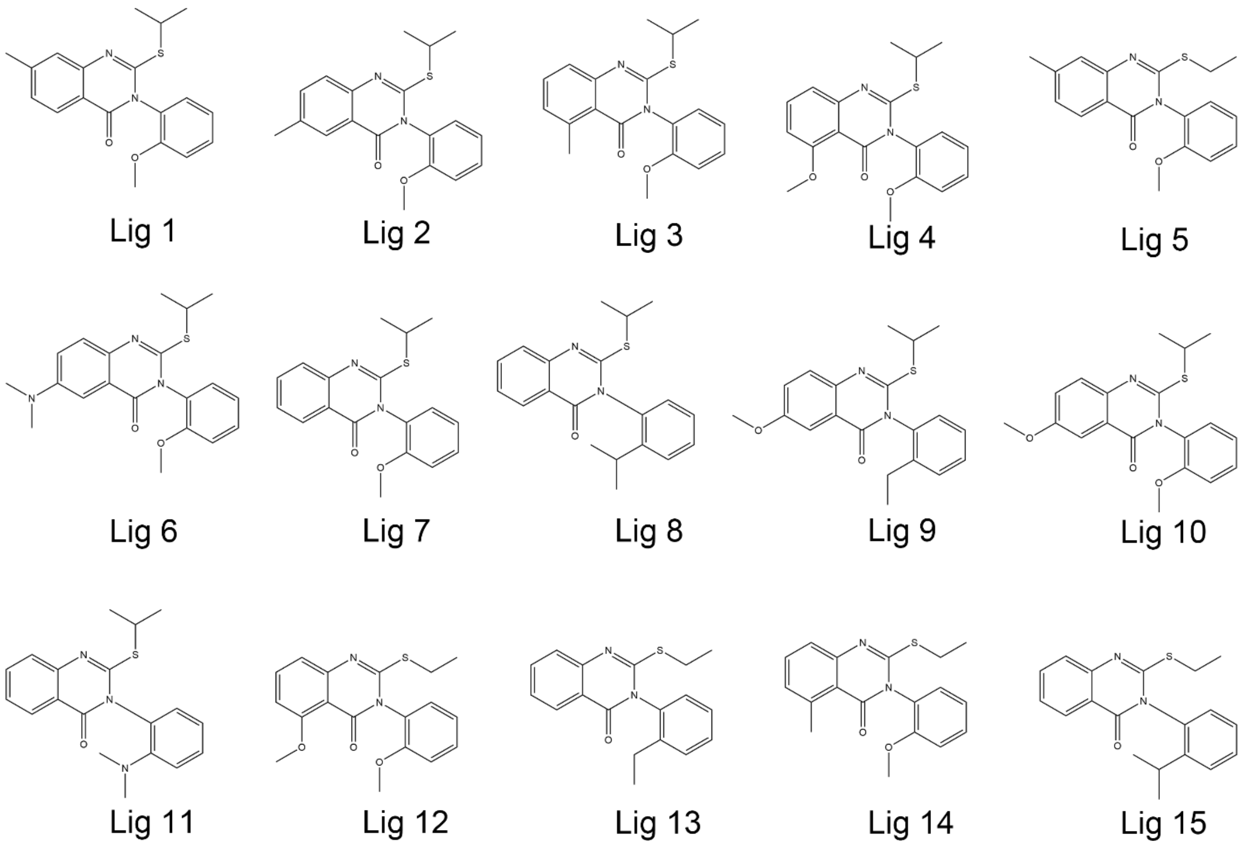

Figure S2. Structure of Lig1-15.

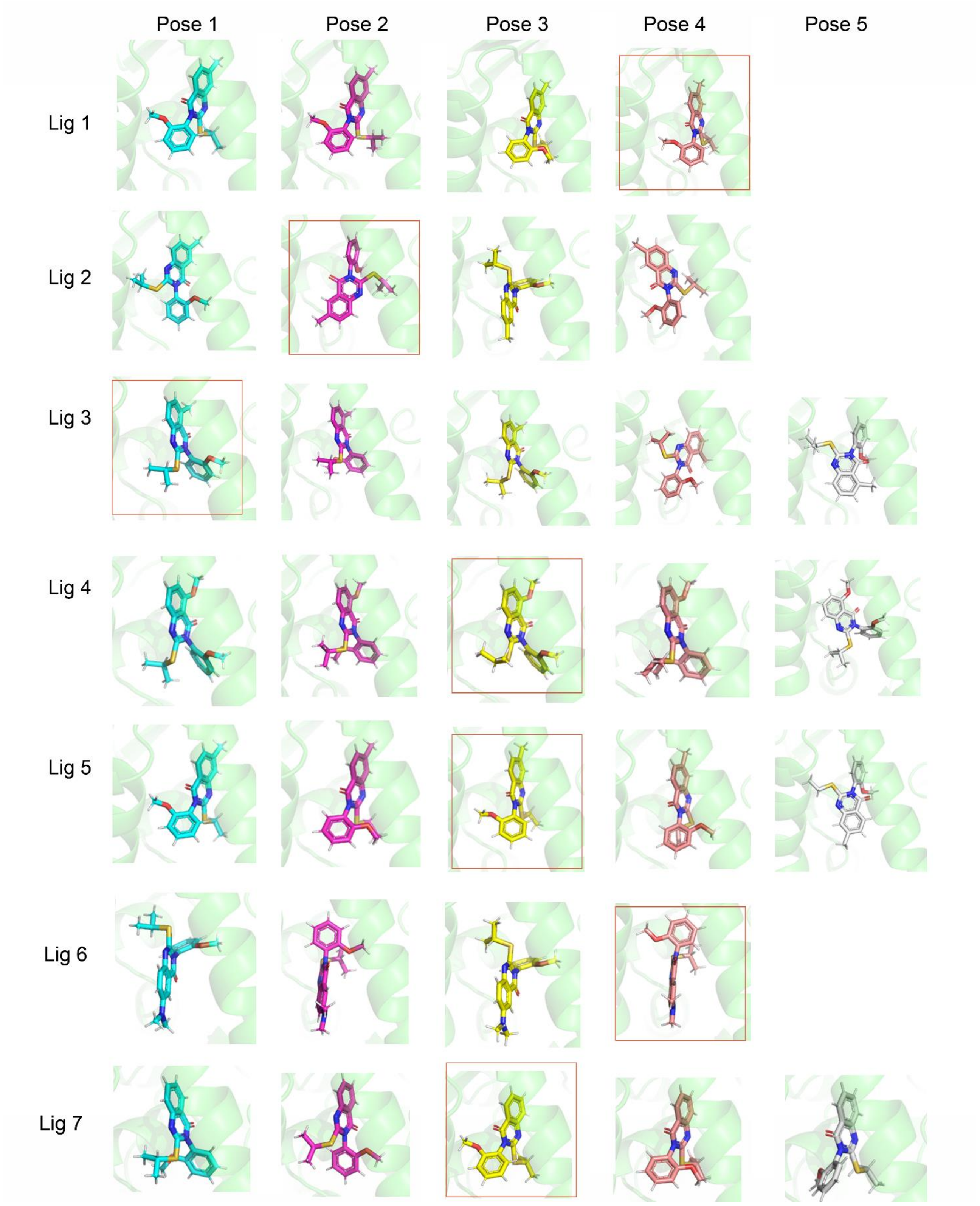

Pose 1
Pose 2
Pose 3
Pose 4
Pose 5
Lig 1
Lig 2
Lig 3
Lig 4
Lig 5
Lig 6
Lig 7

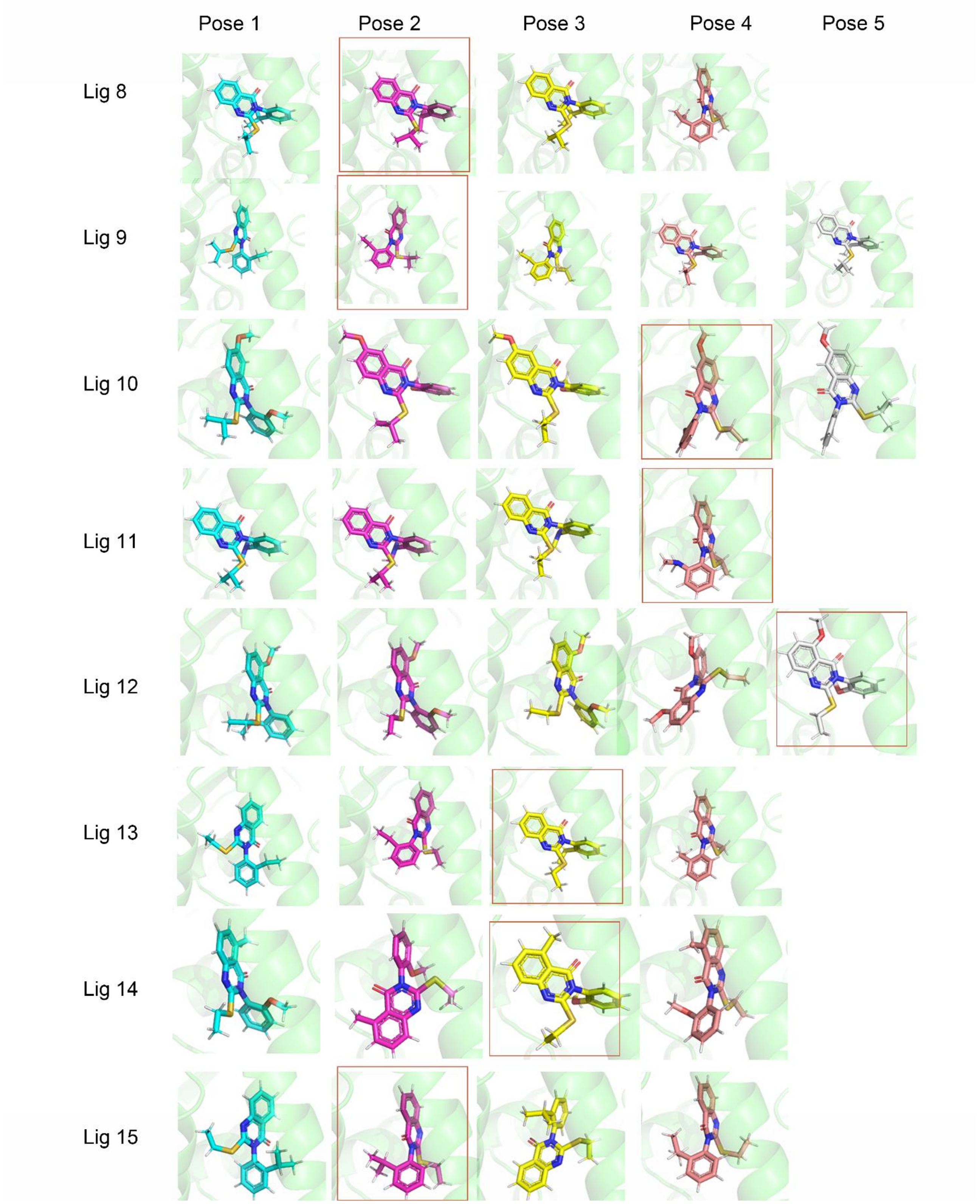

Figure S3. Docking poses of AfDHODH and Lig1-15, the red rectangle shows the RMSD stabilization results in the MD simulation.

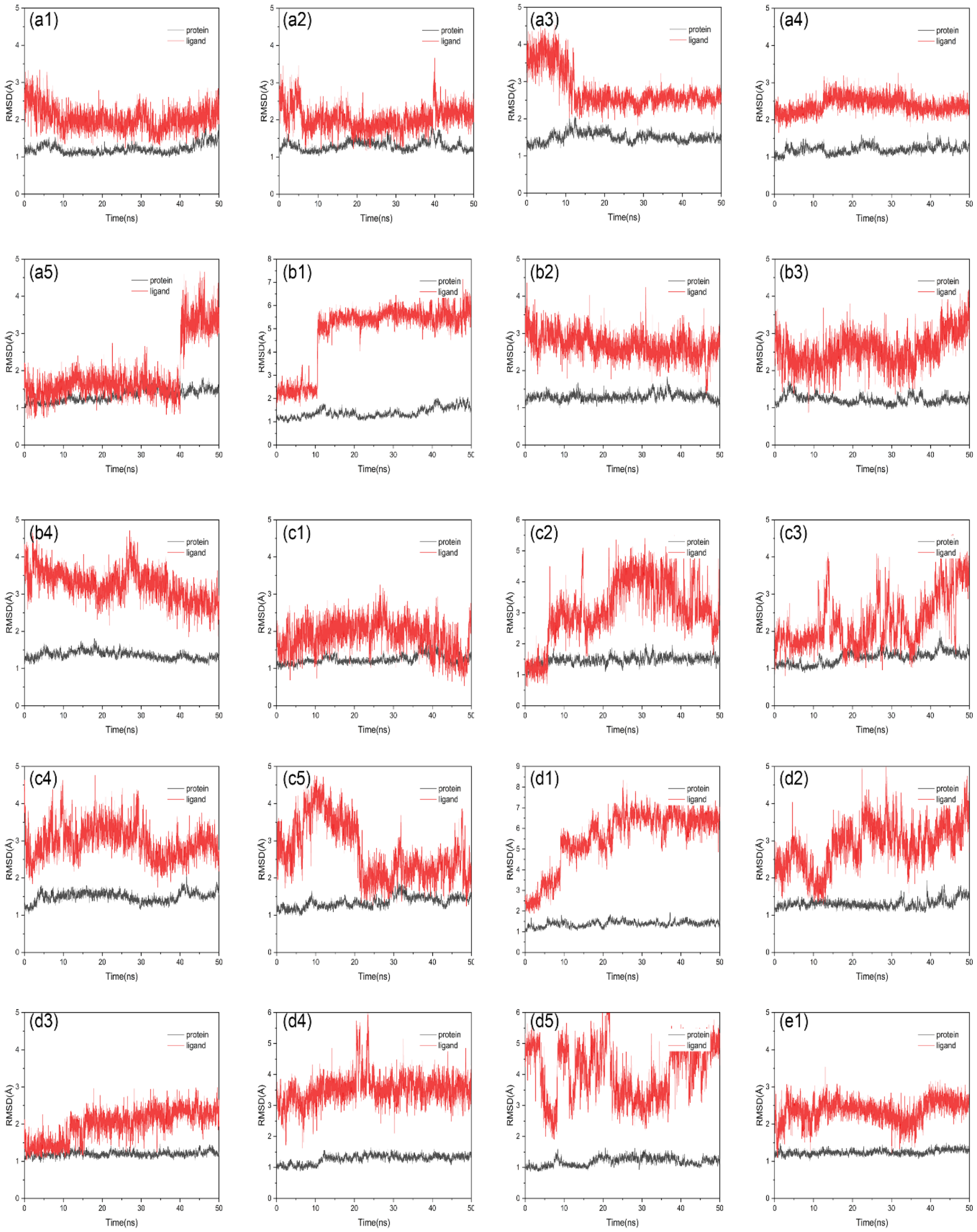
(a1)
(a2)
(a3)
(a4)
(a5)
(b1)
(b2)
(b3)
(b4)
(c1)
(c2)
(c3)
(c4)
(c5)
(d1)
(d2)
(d3)
(d4)
(d5)
(e1)
protein
ligand
RMSD(Å)
Time(ns)

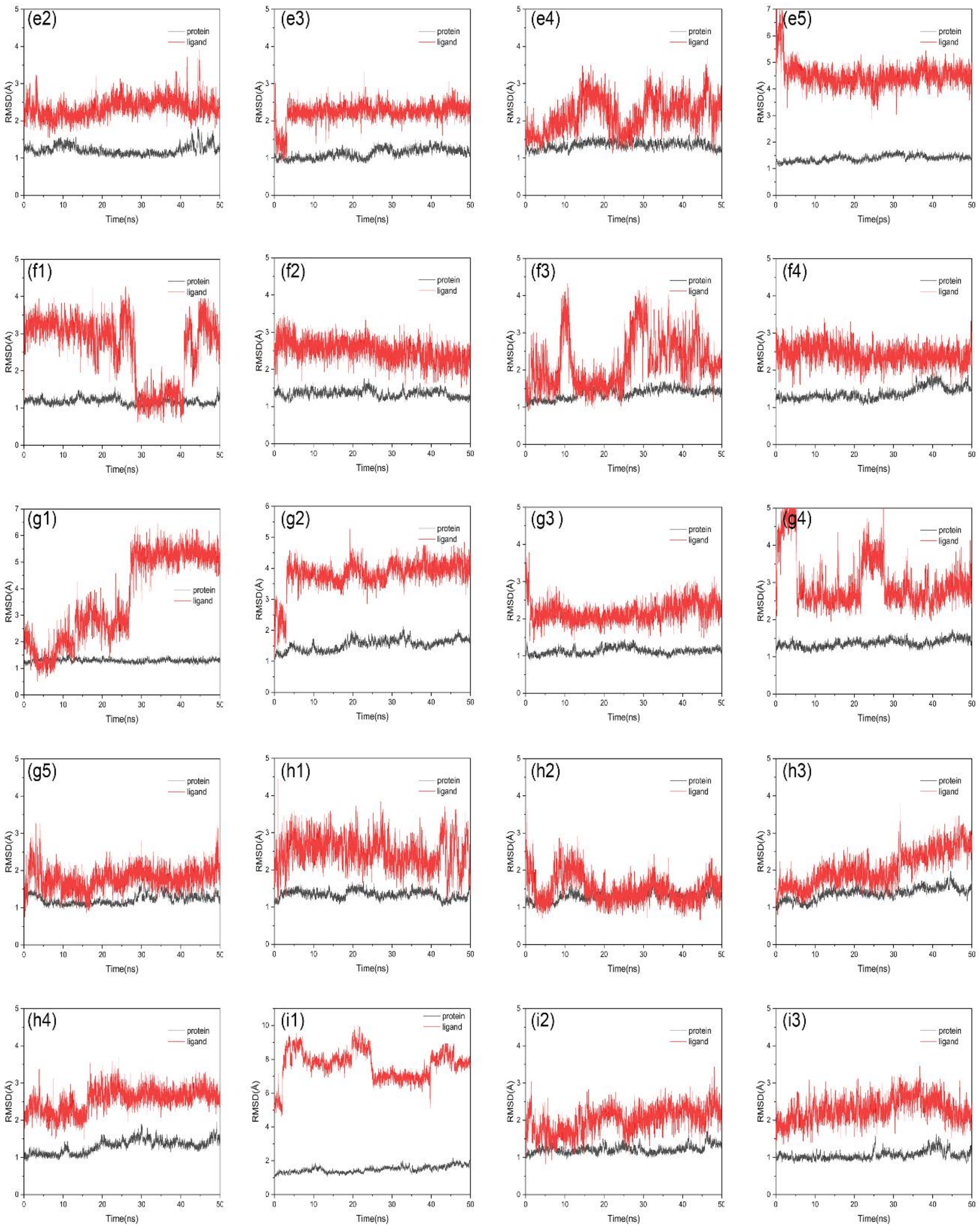
(e2)
(e3)
(e4)
(e5)
(f1)
(f2)
(f3)
(f4)
(g1)
(g2)
(g3)
(g4)
(g5)
(h1)
(h2)
(h3)
(h4)
(i1)
(i2)
(i3)
protein
ligand
RMSD(A)
Time(ns)
Time(ps)

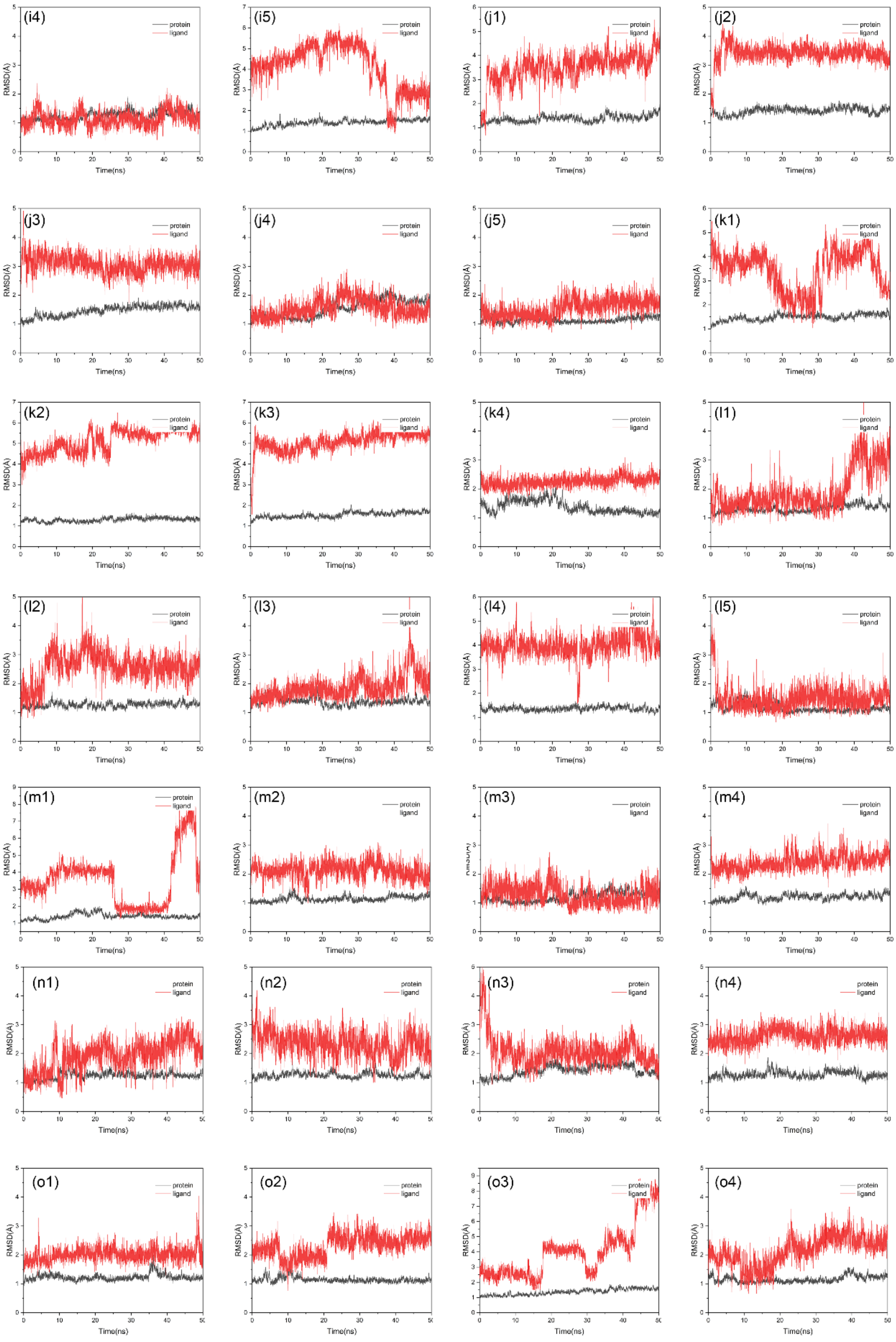

Figure S4. RMSD of Lig1-15 docking pose. (**a**–**o**) Lig1-15.

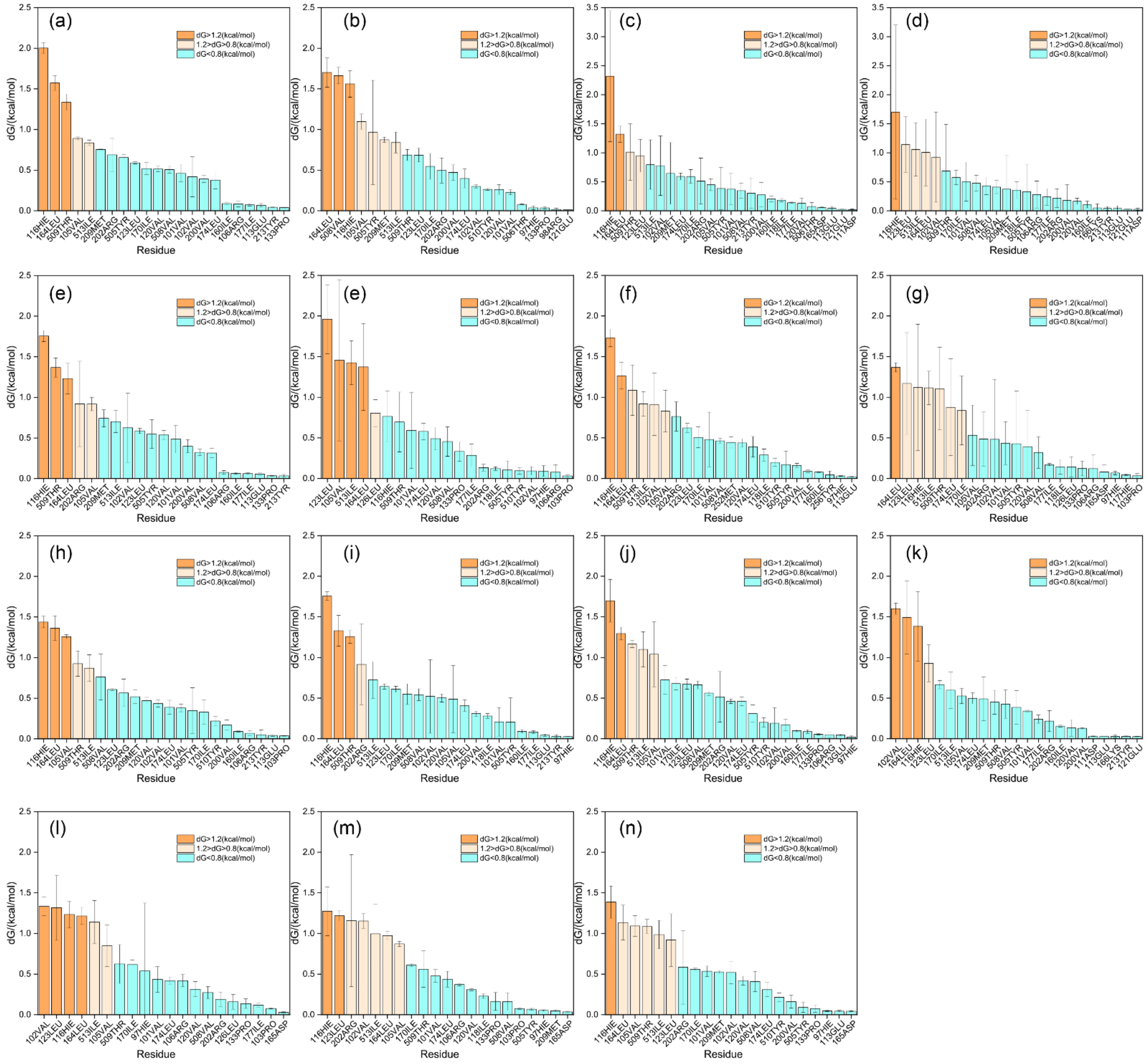

Figure S5. Contribution of residues around the binding pocket to the binding free energy of Lig1-15. (**a**–**n**) Lig1-15.

Table S1. Results and standard errors of different calculation methods for free-binding energy.

Ligand	IC50 (nM)	dGexp (kCal/mol)	dH_avg (kCal/mol)	dIE_3S_avg (kCal/mol)	dGcal_avg (kCal/mol)	MMGBSA (kCal/mol)	Dock Top
olorofim	44	10.0934	21.9811 ± 1.7065	−4.2044 ± 0.1336	17.7768 ± 1.8111	−47.5409 ± 0.9766	/
1	1460	8.006677	15.3472 ± 1.4523	−2.44 ± 0.0631	12.9072 ± 1.3892	−34.7511 ± 1.5935	4
2	2830	7.61231	16.0261 ± 1.9488	−3.1036 ± 0.5803	12.9225 ± 1.3685	−35.7237 ± 1.2178	2
3	4520	7.333305	16.5762 ± 1.7075	−4.0548 ± 0.1412	12.5214 ± 1.8487	−34.3117 ± 2.1876	1
4	4590	7.324148	15.0475 ± 2.5999	−3.706 ± 0.5509	11.3415 ± 2.6517	−33.0235 ± 2.1511	3
5	5400	7.227309	14.2137 ± 0.6476	−2.4303 ± 0.2382	11.7834 ± 0.6224	−33.7768 ± 0.8174	3
6	10,100	6.854216	14.5164 ± 2.1977	−2.5608 ± 0.4795	11.9556 ± 2.1128	−36.169 ± 2.7339	4
7	11,600	6.771707	14.6783 ± 0.988	−2.7444 ± 0.384	11.9339 ± 1.1435	−29.7821 ± 4.3938	3
8	12,200	6.741657	14.4211 ± 2.1049	−2.8216 ± 0.2309	11.5994 ± 2.319	−32.1365 ± 1.4816	2
9	17700	6.519919	13.4676 ± 0.6086	−2.1266 ± 0.2868	11.341 ± 0.7226	−33.699 ± 1.6911	2
10	19,600	6.459161	14.1476 ± 0.9347	−2.6356 ± 0.191	11.512 ± 0.7565	−34.5345 ± 1.5803	4
11	21,200	6.412403	14.4598 ± 1.1562	−2.1972 ± 0.2067	12.2626 ± 0.9516	−35.0261 ± 1.304	4
12	26,900	6.270513	16.1021 ± 1.9333	−5.2977 ± 0.2973	10.8044 ± 2.2307	−33.699 ± 1.6911	5
13	36,200	6.093581	14.1786 ± 0.4243	−2.7391 ± 0.4551	11.4395 ± 0.8	−32.4528 ± 1.448	3
14	43,700	5.981386	14.6509 ± 0.4301	−3.4088 ± 0.1629	11.2421 ± 0.2672	−33.7687 ± 1.9116	3
15	46,100	5.949528	13.5685 ± 0.2503	−2.4252 ± 0.6246	11.1433 ± 0.5562	−35.0389 ± 1.8391	2

Table S2. Results of Free-binding energy for virtual screening.

Ligand	dH (kCal/mol)	dIE_3S (kCal/mol)	dGcal (kCal/mol)	Docking Score	Sim *	Virtual Screening Type
ZINC000408998727	25.2791	−3.9700	21.3091	−10.956	0.126	receptor based
CHEMBL456365	24.2608	−4.2792	19.9816	−11.062	0.208	receptor based
CHEMBL1773415	24.1964	−4.4988	19.6976	−11.237	0.150	receptor based
CHEMBL457850	24.3355	−5.2712	19.0643	−11.111	0.145	receptor based
CHEMBL4227702	23.1802	−4.2492	18.9310	−10.109	0.147	receptor based
CHEMBL1089710	22.7665	−3.9934	18.7731	−10.974	0.106	receptor based
CHEMBL2178724	24.2648	−6.2795	17.9853	−10.019	0.127	receptor based
ZINC000257286719	22.3707	−4.4291	17.9416	−10.845	0.143	receptor based
CHEMBL4100119	21.4668	−3.5967	17.8701	−10.531	0.123	receptor based
CHEMBL1089710	23.1553	−5.3512	17.8041	−11.460	0.106	receptor based
CHEMBL168811	21.6381	−3.8547	17.7834	−10.636	0.116	receptor based
CHEMBL1514025	22.0281	−4.2868	17.7413	−10.044	0.151	receptor based
ZINC000009641177	21.3661	−3.6825	17.6836	−10.836	0.236	receptor based
ZINC000067300707	21.5464	−3.9062	17.6402	−11.357	0.197	receptor based
ZINC000009640749	20.2565	−2.8168	17.4397	−10.941	0.122	receptor based
CHEMBL5089783	22.0762	−4.7656	17.3106	−10.016	0.154	receptor based
ZINC000002691260	20.6070	−3.4168	17.1902	−10.808	0.236	receptor based
CHEMBL202906	21.1268	−3.9698	17.1570	−10.412	0.118	receptor based
ZINC000067301214	21.4859	−4.3381	17.1478	−11.170	0.148	receptor based
ZINC000067301018	21.2853	−4.1764	17.1089	−10.947	0.277	receptor based
ZINC000067300621	21.0244	−3.9770	17.0474	−10.996	0.172	receptor based
CHEMBL1509241	21.5991	−4.6284	16.9708	−10.184	0.092	receptor based
ZINC67300323	19.7742	−3.3980	16.3762	−10.657	0.191	receptor based
CHEMBL468176	20.0583	−1.4102	18.6481	−8.743	0.144	ligand-based 3d
ZINC20897666	22.6537	−4.8417	17.8120	−8.268	0.197	ligand-based 3d
ZINC000329326969	20.4952	−2.9187	17.5765	−8.569	0.238	ligand-based 3d
ZINC000263640002	21.8250	−4.3293	17.4957	−9.020	0.200	ligand-based 3d
ZINC65025513	19.2842	−3.6841	15.6001	−7.557	0.252	ligand-based 2d
ZINC1774881	17.9638	−2.9108	15.0530	−7.845	0.303	ligand-based 2d
ZINC7604321	17.6314	−2.9776	14.6538	−7.448	0.326	ligand-based 2d
ZINC1774881	16.3427	−2.2120	14.1307	−7.845	0.303	ligand-based 2d

*: Sim of Morgan Fingerprints, compared with Olorofim.

Table S3. 3D ligand-based virtual screen result.

Dock Top	dH	dIE_3S	dGcal	Docking Score	Shape sim (3D)	Name
1	21.825	−4.3293	17.4957	−9.02014	0.507	ZINC000263640002
2	21.6267	−4.6561	16.9706	−9.906	0.459	CHEMBL3314028
3	22.564	−8.3126	14.2514	−9.398	0.474	CHEMBL163119
4	18.16	−4.3626	13.7974	−8.97491	0.517	Chemdiv L878-2159
5	20.4697	−5.7799	14.6898	−8.863	0.482	CHEMBL4457849
6	20.3794	−4.978	15.4014	−8.84902	0.477	Chemdiv F531-0104
7	17.6418	−3.7192	13.9226	−8.831	0.457	CHEMBL4438816
8	20.0583	−1.4102	18.6481	−8.743	0.466	CHEMBL468176
9	16.227	−3.5998	12.6272	−8.729	0.489	CHEMBL467557
10	22.0008	−5.4208	16.58	−8.67556	0.477	Chemdiv 4708-0001
11	24.3225	−4.8681	19.4544	−8.65436	0.453	Chemdiv F057-0604
12	20.4952	−2.9187	17.5765	−8.56918	0.513	ZINC000329326969
13	19.7989	−6.115	13.6839	−8.404	0.51	ZINC000065097913
14	21.2702	−4.3507	16.9195	−8.361	0.51	Chemdiv L878-2154
15	19.9025	−4.3579	15.5446	−8.346	0.515	Chemdiv K807-0746
16	13.4937	−4.41	9.0837	−8.333	0.529	ZINC000020798392
17	20.197	−3.6996	16.4974	−8.292	0.534	ZINC000003104289
18	22.6537	−4.8417	17.812	−8.268	0.509	Chemdiv D349-2424
19	17.3286	−2.5849	14.7437	−8.265	0.57	ZINC000426661383

Table S4. 2D ligand-based virtual screen result.

num	dHcal	dIE_3S	dGcal	Glide Score	sim	IUPAC Name
1	13.5995	−3.521	10.0785	−7.989	0.447	N-(4-methylphenyl)-2-oxo-2-(2-phenylindolizin-3-yl)acetamide
2	13.7523	−3.12	10.6323	−7.989	0.447	N-(4-methylphenyl)-2-oxo-2-(2-phenylindolizin-3-yl)acetamide
3	13.6108	−3.4512	10.1596	−8.057	0.428	4-[2-oxo-2-(2-phenylindolizin-3-yl)acetamido]benzamide
4	13.6633	−2.4905	11.1728	−8.638	0.427	2-oxo-N-phenyl-2-(2-phenylindolizin-3-yl)acetamide
5	16.4007	−2.9098	13.4909	−8.894	0.397	N-(3-chlorophenyl)-2-oxo-2-(2-phenylindolizin-3-yl)acetamide
6	15.3837	−4.2864	11.0973	−8.939	0.39	N-(3-acetylphenyl)-2-oxo-2-(2-phenylindolizin-3-yl)acetamide
7	15.0112	−3.593	11.4182	−8.097	0.367	N-(2-chlorophenyl)-2-oxo-2-(2-phenylindolizin-3-yl)acetamide
8	9.846	−1.8144	8.0316	−7.885	0.362	N-methyl-2-oxo-2-(2-phenylindolizin-3-yl)acetamide
9	13.9486	−3.0165	10.9321	−7.618	0.359	N-(2-methoxyphenyl)-2-oxo-2-(2-phenylindolizin-3-yl)acetamide
10	12.0487	−3.2416	8.8071	−7.648	0.358	N-ethyl-2-oxo-2-(2-phenylindolizin-3-yl)acetamide
11	12.5217	−2.1739	10.3478	−7.606	0.358	2-oxo-2-(2-phenylindolizin-3-yl)-N-(1,3-thiazol-2-yl)acetamide
12	14.6912	−3.6807	11.0105	−6.755	0.358	2-oxo-2-(2-phenylindolizin-3-yl)-N-(1,3-thiazol-2-yl)acetamide
13	9.121	−2.5509	6.5701	−7.824	0.356	N-(naphthalen-1-yl)-2-oxo-2-(2-phenylindolizin-3-yl)acetamide
14	11.2818	−3.1692	8.1126	−7.381	0.354	N-(2-hydroxyethyl)-2-oxo-2-(2-phenylindolizin-3-yl)acetamide
15	14.2345	−1.8254	12.4091	−7.352	0.354	N-butyl-2-oxo-2-(2-phenylindolizin-3-yl)acetamide
16	9.4866	−2.0033	7.4833	−7.845	0.354	N-(2-hydroxyethyl)-2-oxo-2-(2-phenylindolizin-3-yl)acetamide
17	14.3017	−2.4824	11.8193	−7.682	0.35	N-tert-butyl-2-oxo-2-(2-phenylindolizin-3-yl)acetamide
18	12.857	−3.6593	9.1977	−9.336	0.344	N-benzyl-2-oxo-2-(2-phenylindolizin-3-yl)acetamide
19	17.06	−3.0706	13.9894	−8.065	0.342	N-[(4-methoxyphenyl)methyl]-2-oxo-2-(2-phenylindolizin-3-yl)acetamide
20	13.6039	−2.0808	11.5231	−8.932	0.338	N-cyclohexyl-2-oxo-2-(2-phenylindolizin-3-yl)acetamide
21	9.4866	−2.0033	7.4833	−7.637	0.334	1-(4-methylpiperazin-1-yl)-2-(2-phenylindolizin-3-yl)ethane-1,2-dione
22	11.1616	−3.7125	7.4491	−6.165	0.334	1-(4-methylpiperazin-1-yl)-2-(2-phenylindolizin-3-yl)ethane-1,2-dione
23	10.4443	−2.1857	8.2586	−7.535	0.326	2-oxo-N-[(oxolan-2-yl)methyl]-2-(2-phenylindolizin-3-yl)acetamide
24	17.6314	−2.9776	14.6538	−7.448	0.326	2-oxo-N-[(oxolan-2-yl)methyl]-2-(2-phenylindolizin-3-yl)acetamide
25	12.0601	−2.5593	9.5008	−8.241	0.317	1-(azepan-1-yl)-2-(2-phenylindolizin-3-yl)ethane-1,2-dione

26	14.1564	−2.5685	11.5879	−7.631	0.316	1-(morpholin-4-yl)-2-(2-phenylindolizin-3-yl)ethane-1,2-dione
27	16.3427	−2.212	14.1307	−7.845	0.303	N-ethyl-2-oxo-N-phenyl-2-(2-phenylindolizin-3-yl)acetamide
28	17.9638	−2.9108	15.053	−7.845	0.303	N-ethyl-2-oxo-N-phenyl-2-(2-phenylindolizin-3-yl)acetamide
29	15.9025	−4.1945	11.708	−7.167	0.287	3-fluoro-N-[2-(4-phenylpiperazin-1-yl)pyrimidin-5-yl]benzamide
30	17.8483	−5.7951	12.0532	−8.143	0.272	2-chloro-N-[2-(4-phenylpiperazin-1-yl)pyrimidin-5-yl]benzamide
31	17.2735	−4.6838	12.5897	−7.896	0.276	2,6-difluoro-N-[2-(4-phenylpiperazin-1-yl)pyrimidin-5-yl]benzamide
32	17.6747	−3.8869	13.7878	−7.275	0.258	N-(4-fluorophenyl)-3-[2-(piperidin-1-yl)pyrimidin-5-yl]benzamide
33	19.2842	−3.6841	15.6001	−7.557	0.252	N-(4-fluorophenyl)-4-[2-(piperidin-1-yl)pyrimidin-5-yl]benzamide
34	17.3285	−3.6829	13.6456	−8.347	0.268	N-(4-fluorophenyl)-2,5-dimethyl-1-[6-(pyrrolidin-1-yl)pyridin-3-yl]-1H-pyrrole-3-carboxamide
35	14.3298	−3.6644	10.6654	−7.602	0.26	2-(2,5-dimethyl-1-phenyl-1H-pyrrol-3-yl)-N-(4-ethylphenyl)-2-oxoacetamide
36	14.5931	−3.5299	11.0632	−8.027	0.251	2-[1-(4-chlorophenyl)-2,5-dimethyl-1H-pyrrol-3-yl]-2-oxo-N-phenylacetamide

Table S5. Receptor-based virtual screen result.

num	dH	dIE_3S	dGcal	Docking Score	sim (2D)	Name
1	25.2791	−3.97	21.3091	−10.956	0.1263	ZINC000408998727
2	24.2608	−4.2792	19.9816	−11.062	0.2083	CHEMBL456365
3	24.1964	−4.4988	19.6976	−11.237	0.15	CHEMBL1773415
4	24.3355	−5.2712	19.0643	−11.111	0.1458	CHEMBL457850
5	23.1802	−4.2492	18.931	−10.109	0.1471	CHEMBL4227702
6	22.7665	−3.9934	18.7731	−10.974	0.1067	CHEMBL1089710
7	23.6307	−5.1052	18.5255	−10.184	0.0928	CHEMBL1509241
8	24.2648	−6.2795	17.9853	−10.019	0.1277	*CHEMBL2178724*
9	22.3707	−4.4291	17.9416	−10.845	0.1429	ZINC000257286719
10	21.4668	−3.5967	17.8701	−10.531	0.1239	CHEMBL4100119
11	23.1553	−5.3512	17.8041	−11.46	0.1067	CHEMBL1089710
12	21.6381	−3.8547	17.7834	−10.636	0.1163	CHEMBL168811
13	22.0281	−4.2868	17.7413	−10.044	0.1515	CHEMBL1514025
14	21.3661	−3.6825	17.6836	−10.836	0.2366	ZINC000009641177
15	21.5464	−3.9062	17.6402	−11.357	0.1978	ZINC000067300707
16	20.2565	−2.8168	17.4397	−10.941	0.1226	ZINC000009640749
17	22.0762	−4.7656	17.3106	−10.016	0.1546	CHEMBL5089783
18	20.607	−3.4168	17.1902	−10.808	0.2366	ZINC000002691260
19	21.1268	−3.9698	17.157	−10.412	0.1183	CHEMBL202906
20	21.4859	−4.3381	17.1478	−11.17	0.1485	ZINC000067301214
21	21.2853	−4.1764	17.1089	−10.947	0.2771	ZINC000067301018
22	21.0244	−3.977	17.0474	−10.996	0.172	ZINC000067300621
23	23.4807	−6.4443	17.0364	−10.771	0.1667	CHEMBL3319084
24	19.4743	−2.5011	16.9732	−10.004	0.1485	CHEMBL1593840
25	21.3178	−4.6181	16.6997	−10.019	0.1429	CHEMBL4461005

26	20.3513	−3.7065	16.6448	−10.657	0.191	ZINC000067300323
27	21.8073	−5.3986	16.4087	−11.431	0.0972	CHEMBL1982212
28	20.8957	−4.6836	16.2121	−10.847	0.1486	ZINC000005502765
29	19.7644	−3.5559	16.2085	−10.992	0.1667	CHEMBL3890389
30	18.993	−2.9829	16.0101	−11.264	0.2584	ZINC000067316877

Table S6. Alanine scanning binding pocket within 5 Å of Olorofim's binding free energy. All units are given in kcal/mol.

Mutation	dVDW	dEEL	dGB	dNP	dH	dIE_3S	dG
90THR	0.1844	−0.0324	−0.0005	0.0102	0.1618	−0.0312	0.1306
97HIE	1.5907	0.0759	−0.2451	0.1171	1.5386	−0.2987	1.2399
101VAL	2.0131	0.0026	−0.2144	0.1852	1.9867	−0.3726	1.614
102VAL	0.4203	0.0558	−0.1271	0.0276	0.3766	−0.1052	0.2715
105VAL	1.7312	0.043	−0.2702	0.1649	1.669	−0.4146	1.2544
113GLU	0.0769	0.257	−0.2307	0.0002	0.1034	−0.0052	0.0982
116HIE	2.2578	0.2736	−0.5204	0.0166	2.0276	−0.379	1.6486
118ILE	0.4109	−0.0203	0.0503	0.0125	0.4535	−0.0506	0.4029
120VAL	0.4175	−0.0098	0.0536	0.0101	0.4715	−0.0475	0.424
123LEU	1.662	−0.0452	−0.1615	0.1381	1.5934	−0.244	1.3494
126LEU	2.0417	0.0247	−0.4041	0.2136	1.876	−0.4785	1.3974
131LEU	0.1169	0.0167	−0.0405	0.0067	0.0999	−0.0609	0.0389
132HIE	0.046	0.0113	0.0002	0.0004	0.0579	−0.0078	0.0502
133PRO	0.9853	0.0199	−0.0469	0.0525	1.0108	−0.2741	0.7367
164LEU	1.8229	0.1073	−0.3644	0.1915	1.7574	−0.3399	1.4174
170ILE	0.3466	−0.0082	0.047	0.0229	0.4084	−0.0172	0.3912
217LEU	0.2659	0.0014	−0.0256	0.0147	0.2565	−0.0098	0.2466
200VAL	0.3457	−0.0374	0.043	0.0327	0.3842	−0.0163	0.3679
202ARG	0.6498	0.2972	−0.294	0.0505	0.7036	−0.0542	0.6494
209MET	0.94	0.2882	−0.3142	0.1205	1.0345	−0.3054	0.7291
213TYR	0.0464	0.0074	−0.0079	0	0.0458	−0.0009	0.0449
505TYR	0.282	−0.0071	−0.0185	0.0176	0.2741	−0.0238	0.2503
508VAL	0.4884	−0.0184	−0.0414	0.0433	0.472	−0.104	0.368
509THR	1.5797	0.305	−0.4156	0.0931	1.5622	−0.3078	1.2544
513ILE	1.598	−0.0144	−0.1086	0.1807	1.6558	−0.255	1.4008
TOTAL	22.3201	1.5936	−3.6573	1.7233	21.9811	−4.2044	17.7768

Table S7. Alanine scanning binding pocket within 5 Å of Lig1's binding free energy. All units are given in kcal/mol.

Mutation	dVDW	dEEL	dGB	dNP	dH	dIE_3S	dG
116HIE	2.6177	0.2665	−0.49965	0.0548	2.4393	−0.43645	2.00285
164LEU	1.71815	−0.03615	−0.0793	0.1669	1.7697	−0.19625	1.57345
509THR	1.49925	0.12305	−0.1621	0.07175	1.532	−0.1961	1.3359
105VAL	1.2795	0.05525	−0.3073	0.13035	1.15785	−0.2662	0.89165
513ILE	0.95145	−0.01185	−0.09015	0.10795	0.95745	−0.12205	0.8354
209MET	1.16355	0.26185	−0.37625	0.15685	1.20605	−0.4508	0.75525
202ARG	0.6965	0.4274	−0.4039	0.05385	0.774	−0.0863	0.6877
505TYR	0.7285	−0.02045	−0.05555	0.07355	0.72605	−0.07095	0.6551
123LEU	0.59515	−0.0272	0.0027	0.0613	0.632	−0.04165	0.59035
170ILE	0.4838	−0.00125	0.0297	0.04315	0.55545	−0.03615	0.5193
120VAL	0.5473	0.0026	0.04115	0.01695	0.6081	−0.09465	0.51345
508VAL	0.58485	−0.00885	−0.04	0.03535	0.57145	−0.066	0.50545
101VAL	0.60355	0.00995	−0.1144	0.0752	0.5744	−0.11515	0.45925
102VAL	0.664	0.0509	−0.14055	0.0569	0.63125	−0.21335	0.4179
200VAL	0.36175	−0.01455	0.0252	0.039	0.4114	−0.01965	0.39175
174LEU	0.35915	−0.02155	0.02415	0.02905	0.3909	−0.01335	0.37755
160ILE	0.07275	−0.0094	0.0255	0	0.08895	−0.0006	0.08835
106ARG	0.07475	0.21485	−0.1981	0.0004	0.09195	−0.0078	0.08415
177ILE	0.0662	−0.0112	0.0181	0.00005	0.07325	−0.0006	0.07265
113GLU	0.08295	−0.0532	0.04195	0.00115	0.0728	−0.0053	0.0675
213TYR	0.0416	0.0121	−0.00995	0	0.0437	−0.0004	0.0433
133PRO	0.03695	−0.02365	0.02585	0	0.03915	−0.0002	0.03895
TOTAL	15.22935	1.18515	−2.2429	1.1745	15.34715	−2.43995	12.9072

Table S8. Alanine scanning binding pocket within 5 Å of Lig2's binding free energy. All units are given in kcal/mol.

Mutation	dVDW	dEEL	dGB	dNP	dH	dIE_3S	dG
TOTAL	15.16995	2.652	−2.91765	1.1219	16.0261	−3.1036	12.9225
164LEU	2.15415	−0.0413	−0.18105	0.23375	2.16565	−0.3824	1.78325
116HIE	1.93415	0.3312	−0.44465	0.065	1.8856	−0.22965	1.65595
508VAL	1.90985	−0.0257	−0.0686	0.05565	1.8712	−0.2364	1.6348
105VAL	1.29925	0.09175	−0.14475	0.14465	1.3909	−0.26565	1.12525
209MET	1.49705	0.57465	−0.67695	0.1618	1.55655	−0.66585	0.8907
513ILE	0.89525	−0.0269	−0.0485	0.08945	0.9093	−0.14125	0.76805
505TYR	0.8093	0.1028	−0.1517	0.05655	0.8168	−0.0918	0.725
509THR	0.6733	1.3737	−0.8307	0.0513	1.26755	−0.62015	0.6474
123LEU	0.67795	−0.0262	−0.0275	0.07295	0.69725	−0.0605	0.63675
200VAL	0.5584	−0.0414	−0.00865	0.05725	0.56555	−0.04425	0.5213
170ILE	0.47065	−0.00235	0.0169	0.0406	0.52585	−0.0291	0.49675
202ARG	0.4023	0.437	−0.40205	0.0146	0.4519	−0.0251	0.4268
174LEU	0.3682	−0.00275	−0.016	0.0278	0.3773	−0.01785	0.35945
102VAL	0.3559	0.0042	−0.0013	0.0048	0.36355	−0.0542	0.30935
120VAL	0.35535	0.0143	−0.00735	0.00795	0.3702	−0.0788	0.2914
510TYR	0.2236	−0.12665	0.1786	−0.00005	0.27545	−0.006	0.26945
101VAL	0.43165	−0.02065	−0.0651	0.03885	0.38475	−0.153	0.23175
506THR	0.07895	0.0297	−0.02715	−0.001	0.0805	−0.0004	0.0801
133PRO	0.0258	−0.01075	0.0127	0	0.02775	−0.00005	0.0277
97HIE	0.0267	0.0261	−0.0286	0	0.02415	−0.00075	0.0234
121GLU	0.01105	0.00345	−0.0038	0	0.0108	−0.0002	0.0106
98ARG	0.01115	−0.0122	0.00855	0	0.00755	−0.00025	0.0073

Table S9. Alanine scanning binding pocket within 5 Å of Lig3's binding free energy. All units are given in kcal/mol.

Mutation	dVDW	dEEL	dGB	dNP	dH	dIE_3S	dG
116HIE	3.2677	0.093267	−0.38003	0.079733	3.0607	−0.74173	2.318967
164LEU	1.904967	0.1384	−0.44427	0.161433	1.7605	−0.44007	1.320433
509THR	1.2249	0.436267	−0.31213	0.074333	1.4233	−0.4116	1.0117
123LEU	1.119633	−0.03697	−0.04687	0.115233	1.150967	−0.2018	0.949167
513ILE	1.2038	0.02	−0.0751	0.124833	1.273467	−0.47717	0.7963
102VAL	1.021233	0.048667	−0.1006	0.082133	1.0514	−0.27353	0.777867
209MET	0.858933	0.075967	−0.20807	0.086333	0.813133	−0.16973	0.6434
174LEU	0.6628	−0.03957	−0.0387	0.068233	0.6527	−0.06117	0.591533
170ILE	0.6057	−0.01133	−0.00203	0.0602	0.652433	−0.06103	0.5914
202ARG	0.581167	0.044167	−0.06887	0.038367	0.5948	−0.08083	0.513967
105VAL	0.6528	−0.00427	−0.06117	0.0595	0.646733	−0.1968	0.449933
505TYR	0.474633	0.0203	−0.06367	0.043333	0.474633	−0.09013	0.3845
101VAL	0.5463	−0.00703	−0.04177	0.057967	0.555367	−0.1828	0.372567
508VAL	0.6863	0.008767	−0.04857	0.040733	0.687133	−0.34107	0.346067
213TYR	0.3541	0.0512	−0.0856	0.028633	0.348267	−0.045	0.303267
200VAL	0.2864	0.001367	−0.00903	0.0199	0.298567	−0.0238	0.274767
160ILE	0.2061	−0.0108	0.0135	0.008867	0.217633	−0.0146	0.203033
118ILE	0.1398	−0.00363	0.069167	0.002	0.207267	−0.0229	0.184367
177ILE	0.134267	−0.01073	0.017633	0.003533	0.1447	−0.0059	0.1388
120VAL	0.281633	0.001033	−0.00083	0.013233	0.295	−0.16663	0.128367
506THR	0.095733	0.0472	−0.04647	0.0048	0.1012	−0.0281	0.0731
165ASP	0.047733	0.0993	−0.07607	−0.00013	0.0708	−0.01263	0.058167
113GLU	0.0623	−0.07583	0.0605	6.67E−05	0.046967	−0.00333	0.043633
121GLU	0.018533	0.050433	−0.04223	0	0.0267	−0.00163	0.025067
111ASP	0.017967	−0.0206	0.0245	0	0.021833	−0.0008	0.021033
TOTAL	16.45543	0.915567	−1.96677	1.173267	16.5762	−4.0548	12.5214

Table S10. Alanine scanning binding pocket within 5 Å of Lig4's binding free energy. All units are given in kcal/mol.

Mutation	dVDW	dEEL	dGB	dNP	dH	dIE_3S	dG
116HIE	2.491333	−0.12427	−0.16737	0.0845	2.284267	−0.58353	1.700733
123LEU	1.516767	0.0234	−0.16487	0.1397	1.515033	−0.37293	1.1421
513ILE	1.483267	−0.0003	−0.1573	0.172967	1.498633	−0.44183	1.0568
164LEU	1.491033	0.0966	−0.3666	0.125067	1.346133	−0.33993	1.0062
102VAL	1.361467	0.0787	−0.2229	0.1214	1.338633	−0.41547	0.923167
509THR	0.9153	0.184167	−0.1809	0.041133	0.959767	−0.27333	0.686433
170ILE	0.589367	−0.00157	−0.01177	0.050367	0.6264	−0.052	0.5744
101VAL	0.699367	−0.00303	−0.05747	0.0717	0.710533	−0.20697	0.503567
508VAL	0.7631	−0.03333	−0.12407	0.0353	0.641033	−0.1659	0.475133
174LEU	0.501067	−0.0344	−0.0424	0.046367	0.4707	−0.04	0.4307
105VAL	0.597867	−0.00627	−0.05817	0.043333	0.5768	−0.16793	0.408867
209MET	0.5595	0.1261	−0.18027	0.0543	0.559633	−0.18627	0.373367
118ILE	0.3488	−0.0149	0.075233	0.010833	0.419967	−0.06287	0.3571
505TYR	0.369967	−0.02847	−0.00327	0.026867	0.365133	−0.0354	0.329733
106ARG	0.4186	0.05	−0.0682	0.030533	0.4309	−0.15107	0.279833
177ILE	0.254733	−0.001	−0.01217	0.016533	0.258167	−0.0187	0.239467
202ARG	0.268267	−0.00067	−0.01963	0.020867	0.2688	−0.04963	0.219167
200VAL	0.183533	−0.00377	−0.00267	0.015467	0.192567	−0.0112	0.181367
120VAL	0.275133	−0.02897	0.0276	0.006833	0.280533	−0.1166	0.163933
160ILE	0.107533	−0.0055	0.0087	0.0018	0.112533	−0.0039	0.108633
166LYS	0.052867	−0.02573	0.025267	−3.3E−05	0.052367	−0.0029	0.049467
213TYR	0.042167	0.004233	−0.0053	0	0.0411	−0.0007	0.0404
113GLU	0.048633	0.023833	−0.02833	3.33E−05	0.0442	−0.00427	0.039933
121GLU	0.024433	0.0164	−0.0133	0	0.027533	−0.00117	0.026367
111ASP	0.0149	0.0461	−0.03487	0	0.026167	−0.0015	0.024667
TOTAL	15.379	0.337367	−1.785	1.115867	15.04753	−3.706	11.34153

Table S11. Alanine scanning binding pocket within 5 Å of Lig5's binding free energy. All units are given in kcal/mol.

Mutation	dVDW	dEEL	dGB	dNP	dH	dIE_3S	dG
116HIE	2.14622	0.2409	−0.34078	0.04856	2.0949	−0.33976	1.75514
509THR	1.5922	0.1034	−0.16836	0.08538	1.61264	−0.24508	1.36756
164LEU	1.46232	−0.0081	−0.12762	0.13636	1.46294	−0.23126	1.23168
202ARG	0.9909	0.39042	−0.3937	0.08992	1.07748	−0.15766	0.91982
105VAL	1.26228	0.05506	−0.27114	0.1275	1.17364	−0.2563	0.91734
209MET	1.0546	0.29774	−0.39894	0.1407	1.09408	−0.3527	0.74138
513ILE	0.8807	−0.02104	−0.11916	0.09988	0.84042	−0.13826	0.70216
102VAL	0.92126	0.09036	−0.2124	0.07508	0.87428	−0.24824	0.62604
123LEU	0.62132	−0.02608	0.00014	0.06278	0.65814	−0.07048	0.58766
505TYR	0.6325	−0.00618	−0.0718	0.05996	0.61444	−0.06704	0.5474
120VAL	0.55532	−0.00504	0.0538	0.0112	0.61532	−0.07766	0.53766
101VAL	0.65772	0.00028	−0.1153	0.079	0.62172	−0.13604	0.48568
200VAL	0.37184	−0.01348	0.02394	0.03622	0.41858	−0.02138	0.3972
508VAL	0.38236	−0.0245	0.00656	0.02198	0.38642	−0.0624	0.32402
174LEU	0.30432	−0.02456	0.02352	0.0221	0.32538	−0.01074	0.31464
106ARG	0.0704	0.20322	−0.18778	0.00024	0.08612	−0.00894	0.07718
160ILE	0.05172	−0.00862	0.02214	0.00004	0.06524	−0.0002	0.06504
177ILE	0.05732	−0.01028	0.01466	0.00002	0.06172	−0.00048	0.06124
113GLU	0.07146	−0.0355	0.02368	0.00032	0.05998	−0.00488	0.0551
133PRO	0.0341	−0.023	0.02434	0	0.03544	−0.00022	0.03522
213TYR	0.03446	0.00484	−0.0045	0.00006	0.0348	−0.0006	0.0342
TOTAL	14.15532	1.17984	−2.2187	1.0973	14.21368	−2.43032	11.78336

Table S12. Alanine scanning binding pocket within 5 Å of Lig6's binding free energy. All units are given in kcal/mol.

Mutation	dVDW	dEEL	dGB	dNP	dH	dIE_3S	dG
123LEU	2.564867	0.041833	−0.31497	0.180667	2.472367	−0.51193	1.960433
105VAL	1.863067	0.017	−0.25173	0.152133	1.780467	−0.32533	1.455133
513ILE	1.821233	0.012533	−0.225	0.151367	1.760133	−0.3352	1.424933
164LEU	1.7222	0.073967	−0.29773	0.1965	1.694933	−0.32147	1.373467
126LEU	1.087	0.004367	−0.21133	0.1471	1.027133	−0.2226	0.804533
116HIE	1.009167	0.118233	−0.26667	0.055933	0.916567	−0.15213	0.764433
509THR	0.849333	0.112733	−0.17203	0.0625	0.8525	−0.157	0.6955
101VAL	0.768933	−0.0028	−0.08447	0.067033	0.748667	−0.1581	0.590567
174LEU	0.6157	0.0009	−0.0448	0.0601	0.631833	−0.05303	0.5788
120VAL	0.547067	−0.0497	0.0547	0.021167	0.573267	−0.08297	0.4903
508VAL	0.4924	0.017433	−0.00367	0.042633	0.548833	−0.0943	0.454533
133PRO	0.371533	−0.00187	−0.01933	0.0266	0.376933	−0.04483	0.3321
177ILE	0.279833	0.004	0.007167	0.020833	0.311833	−0.02713	0.2847
202ARG	0.1662	−0.07567	0.0462	0.0086	0.145333	−0.01213	0.1332
118ILE	0.075233	−0.0153	0.068967	0.0007	0.129633	−0.0084	0.121233
505TYR	0.1349	−0.00077	−0.00967	0.0057	0.130067	−0.02553	0.104533
510TYR	0.081033	0.023633	−0.0061	3.33E−05	0.098533	−0.00377	0.094767
102VAL	0.095	−0.0078	0.011233	0.001833	0.100267	−0.0068	0.093467
97HIE	0.1323	0.0018	−0.0359	0.002467	0.1006	−0.00963	0.090967
106ARG	0.096367	−0.0365	0.025433	−0.00063	0.084633	−0.0079	0.076733
103PRO	0.0246	−0.0033	0.0105	0	0.031867	−0.00063	0.031233
TOTAL	14.79797	0.234733	−1.7192	1.203267	14.5164	−2.56083	11.95557

Table S13. Alanine scanning binding pocket within 5 Å of Lig7's binding free energy. All units are given in kcal/mol.

Mutation	dVDW	dEEL	dGB	dNP	dH	dIE_3S	dG
116HIE	2.2532	0.163333	−0.38047	0.0507	2.0867	−0.35443	1.732267
164LEU	1.470367	−0.05877	−0.0902	0.160667	1.482	−0.21773	1.264267
509THR	1.5061	−0.01083	−0.16	0.1179	1.453167	−0.36687	1.0863
513ILE	1.097667	−0.01727	−0.1279	0.129667	1.0821	−0.1645	0.9176
102VAL	1.409933	0.105233	−0.33743	0.1139	1.2916	−0.3797	0.9119
105VAL	1.090367	0.0268	−0.1344	0.0983	1.081033	−0.2516	0.829433
202ARG	0.87	0.349833	−0.35703	0.080067	0.942867	−0.17983	0.763033
123LEU	0.679667	−0.01703	−0.0118	0.068767	0.7195	−0.0964	0.6231
170ILE	0.474567	0.000267	0.028133	0.039967	0.542833	−0.03633	0.5065
101VAL	0.6881	0.020933	−0.11823	0.0817	0.672467	−0.1923	0.480167
508VAL	0.5427	−0.02007	−0.01753	0.0398	0.5448	−0.08117	0.463633
252MET	0.7581	0.113933	−0.2734	0.113833	0.712433	−0.26817	0.444267
120VAL	0.465967	−0.00263	0.033667	0.012933	0.5099	−0.0726	0.4373
174LEU	0.3788	−0.01673	0.0143	0.032933	0.409233	−0.0187	0.390533
118ILE	0.2462	−0.0202	0.0785	0.007267	0.3117	−0.02257	0.289133
510TYR	0.194767	−0.05193	0.0612	0.000933	0.204967	−0.00663	0.198333
505TYR	0.2063	−0.0179	−0.00717	0.011167	0.1923	−0.0215	0.1708
200VAL	0.147833	−0.0035	0.012367	0.008833	0.165433	−0.0051	0.160333
177ILE	0.0829	−0.0139	0.019567	0.0004	0.088967	−0.0014	0.087567
160ILE	0.0639	−0.00943	0.023567	6.67E−05	0.078	−0.00047	0.077533
256TYR	0.053567	0.015967	−0.02277	0.002367	0.049133	−0.00287	0.046267
97HIE	0.0407	0.0885	−0.0964	−3.3E−05	0.032733	−0.00187	0.030867
113GLU	0.037567	−0.08827	0.075133	0	0.0244	−0.00163	0.022767
TOTAL	14.75927	0.536333	−1.7883	1.172133	14.67827	−2.74437	11.9339

Table S14. Alanine scanning binding pocket within 5 Å of Lig8's binding free energy. All units are given in kcal/mol.

Mutation	dVDW	dEEL	dGB	dNP	dH	dIE_3S	dG
164LEU	1.931967	0.118433	−0.41117	0.1808	1.820067	−0.45243	1.367633
123LEU	1.3676	0.019767	−0.12213	0.129367	1.3947	−0.22557	1.169133
116HIE	1.587267	0.113133	−0.21627	0.017633	1.501867	−0.37963	1.122233
513ILE	1.280433	−0.01193	−0.0589	0.138733	1.348333	−0.23283	1.1155
509THR	1.465967	−0.09933	−0.0945	0.133867	1.406033	−0.30087	1.105167
174LEU	0.943667	−0.0025	−0.08873	0.103467	0.955867	−0.07903	0.876833
170ILE	0.846833	−0.00153	−0.0159	0.078233	0.907633	−0.07063	0.837
105VAL	0.719	0.023767	−0.09663	0.056967	0.7031	−0.1715	0.5316
202ARG	0.530333	−0.08127	0.0448	0.0498	0.5437	−0.0583	0.4854
102VAL	0.636033	0.053067	−0.08287	0.058567	0.6648	−0.1818	0.483
101VAL	0.567333	−0.00607	−0.0343	0.061267	0.588233	−0.15307	0.435167
505TYR	0.516767	0.1608	−0.19513	0.036267	0.518667	−0.09003	0.428633
120VAL	0.6293	−0.00233	−0.0454	0.023833	0.605367	−0.2136	0.391767
508VAL	0.507467	−0.00947	−0.0527	0.0425	0.487833	−0.16747	0.320367
177ILE	0.157233	−0.00453	0.019233	0.002733	0.174633	−0.00453	0.1701
118ILE	0.0904	0.000833	0.057467	−3.3E−05	0.1486	−0.00443	0.144167
126LEU	0.143633	−0.00877	0.006567	0.009967	0.151433	−0.00863	0.1428
133PRO	0.116933	−0.01417	0.019433	0.005867	0.128067	−0.00457	0.1235
106ARG	0.144267	−0.05727	0.044633	0.004067	0.135767	−0.01473	0.121033
165ASP	0.077667	0.020233	−0.01257	−0.00213	0.083233	−0.00237	0.080867
97HIE	0.0698	0.034767	−0.03363	0.000567	0.071567	−0.004	0.067567
117HIE	0.041167	−0.01447	0.0206	0	0.0474	−0.00097	0.046433
103PRO	0.031	0.003633	−0.00117	0.000667	0.034167	−0.00063	0.033533
TOTAL	14.40207	0.2348	−1.34927	1.133	14.42107	−2.82163	11.59943

Table S15. Alanine scanning binding pocket within 5 Å of Lig9's binding free energy. All units are given in kcal/mol.

Mutation	dVDW	dEEL	dGB	dNP	dH	dIE_3S	dG
116HIE	1.9106	0.148567	−0.32553	0.0491	1.7827	−0.34507	1.437633
164LEU	1.517133	−0.02657	−0.08247	0.1701	1.578167	−0.21593	1.362233
105VAL	1.653033	0.076267	−0.32967	0.173667	1.573367	−0.31537	1.258
509THR	1.148367	0.032567	−0.14473	0.071867	1.108067	−0.1817	0.926367
513ILE	0.9954	−0.008	−0.052	0.116567	1.051933	−0.18127	0.870667
508VAL	0.9478	−0.01977	−0.01327	0.061667	0.976467	−0.21527	0.7612
123LEU	0.6065	−0.02147	−0.00107	0.069367	0.653367	−0.0451	0.608267
202ARG	0.550467	0.4327	−0.41037	0.0379	0.6107	−0.04507	0.565633
209MET	0.728833	0.032533	−0.1677	0.095	0.688733	−0.17	0.518733
120VAL	0.534033	0.001467	0.018733	0.022633	0.576833	−0.10607	0.470767
102VAL	0.581667	−0.00437	−0.06307	0.050367	0.564633	−0.13	0.434633
174LEU	0.368933	−0.0229	0.0249	0.0319	0.402767	−0.01433	0.388433
101VAL	0.4723	−0.00317	−0.07693	0.054167	0.446433	−0.06687	0.379567
505TYR	0.409433	−0.0454	−0.00357	0.030033	0.390567	−0.04233	0.348233
170ILE	0.308733	−0.00297	0.024033	0.0226	0.352367	−0.0224	0.329967
510TYR	0.2091	−0.05077	0.071667	0	0.23	−0.01097	0.219033
200VAL	0.158833	−0.01133	0.020467	0.010567	0.178567	−0.00647	0.1721
160ILE	0.0745	−0.008	0.026233	0.000233	0.092933	−0.0007	0.092233
106ARG	0.0627	0.162567	−0.15073	0.0001	0.074633	−0.0053	0.069333
213TYR	0.049633	0.000867	0.000867	0.000967	0.052367	−0.00223	0.050133
113GLU	0.051033	−0.01677	0.010767	0.000233	0.045233	−0.00383	0.0414
103PRO	0.030867	−0.0162	0.021967	0.000133	0.036733	−0.0003	0.036433
TOTAL	13.3699	0.629867	−1.60147	1.069167	13.46757	−2.12657	11.341

Table S16. Alanine scanning binding pocket within 5 Å of Lig10's binding free energy. All units are given in kcal/mol.

Mutation	dVDW	dEEL	dGB	dNP	dH	dIE_3S	dG
116HIE	2.291233	0.294233	−0.4975	0.0363	2.1243	−0.3688	1.7555
164LEU	1.571967	−0.05463	−0.1062	0.163467	1.5747	−0.24537	1.329333
509THR	1.539967	0.0959	−0.20533	0.102567	1.533067	−0.2763	1.256767
202ARG	1.084567	0.535333	−0.49863	0.089567	1.2108	−0.2965	0.9143
513ILE	0.856433	−0.0267	−0.08543	0.1036	0.847933	−0.12407	0.723867
123LEU	0.6693	−0.0296	−0.00563	0.071867	0.705967	−0.0631	0.642867
170ILE	0.566167	0.000833	0.0285	0.0465	0.642033	−0.0331	0.608933
209MET	1.007	0.338167	−0.50287	0.140167	0.9825	−0.43297	0.549533
508VAL	0.637	−0.01777	−0.06217	0.0416	0.598667	−0.05687	0.5418
102VAL	0.8712	0.06	−0.1929	0.075867	0.8142	−0.29407	0.520133
120VAL	0.5394	−0.00303	0.040867	0.012333	0.589567	−0.08787	0.5017
105VAL	0.671067	−0.0196	−0.09583	0.0587	0.614333	−0.12687	0.487467
174LEU	0.394633	−0.01967	0.0165	0.032467	0.424067	−0.01817	0.4059
200VAL	0.341767	−0.00553	−0.02317	0.0383	0.351367	−0.04247	0.3089
118ILE	0.242567	−0.02647	0.0819	0.007767	0.3058	−0.0256	0.2802
101VAL	0.301033	0.022133	−0.08067	0.038333	0.280933	−0.0737	0.207233
505TYR	0.269367	−0.00747	−0.0163	0.018533	0.2641	−0.05807	0.206033
160ILE	0.076	−0.0089	0.024133	6.67E−05	0.0913	−0.00057	0.090733
177ILE	0.0774	−0.01207	0.017567	0.0002	0.0831	−0.00093	0.082167
113GLU	0.060867	−0.0494	0.0398	0.000167	0.051433	−0.00837	0.043067
213TYR	0.031133	0.010567	−0.01097	6.67E−05	0.030767	−0.0009	0.029867
97HIE	0.0346	0.0703	−0.07817	0	0.026667	−0.001	0.025667
TOTAL	14.13467	1.146633	−2.2125	1.078433	14.1476	−2.63563	11.51197

Table S17. Alanine scanning binding pocket within 5 Å of Lig11's binding free energy. All units are given in kcal/mol.

Mutation	dVDW	dEEL	dGB	dNP	dH	dIE_3S	dG
116HIE	2.2055	0.098933	−0.29227	0.0488	2.060967	−0.3628	1.698167
164LEU	1.5277	−0.01643	−0.09247	0.1401	1.558967	−0.26423	1.294733
509THR	1.311067	0.092233	−0.12463	0.0672	1.345933	−0.18073	1.1652
513ILE	1.308833	−0.0144	−0.04903	0.141733	1.387133	−0.2876	1.099533
105VAL	1.341367	0.098433	−0.27493	0.128167	1.2931	−0.2538	1.0393
101VAL	0.8417	−0.0024	−0.0567	0.081567	0.864267	−0.13983	0.724433
170ILE	0.627633	−0.00363	0.0534	0.058167	0.7356	−0.05683	0.678767
123LEU	0.701367	−0.01803	−0.00753	0.078433	0.7543	−0.0823	0.672
508VAL	0.680833	−0.01297	0.0427	0.042233	0.752867	−0.0907	0.662167
209MET	0.789233	0.183967	−0.3227	0.0974	0.747933	−0.18683	0.5611
202ARG	0.5276	0.294667	−0.283	0.0395	0.578867	−0.06323	0.515633
120VAL	0.4713	−0.00387	0.057933	0.0073	0.532733	−0.07163	0.4611
174LEU	0.437467	−0.0317	0.0355	0.037267	0.478567	−0.0176	0.460967
505TYR	0.371533	−0.0516	0.0027	0.023	0.345633	−0.03677	0.308867
510TYR	0.189933	−0.0418	0.0593	6.67E−05	0.2076	−0.00647	0.201133
102VAL	0.268067	0.0003	−0.01623	0.0226	0.2747	−0.08293	0.191767
200VAL	0.159633	−0.0121	0.0221	0.0076	0.1773	−0.00523	0.172067
160ILE	0.077567	−0.009	0.030633	0.0003	0.099533	−0.00107	0.098467
177ILE	0.070033	−0.0067	0.0248	0.0002	0.088367	−0.00077	0.0876
133PRO	0.052167	−0.0127	0.015767	0	0.055333	−0.00053	0.0548
106ARG	0.039933	0.139133	−0.12793	0	0.051233	−0.00187	0.049367
113GLU	0.0692	−0.03823	0.017067	0	0.0481	−0.00293	0.045167
97HIE	0.029333	0.027233	−0.0358	0	0.0208	−0.00053	0.020267
TOTAL	14.099	0.659333	−1.32133	1.021633	14.45983	−2.19723	12.2626

Table S18. Alanine scanning binding pocket within 5 Å of Lig12's binding free energy. All units are given in kcal/mol.

Mutation	dVDW	dEEL	dGB	dNP	dH	dIE_3S	dG
102VAL	1.9063	0.1078	−0.171	0.1227	1.965767	−0.36467	1.6011
164LEU	2.2204	0.051667	−0.54823	0.1753	1.899133	−0.40693	1.4922
116HIE	2.7001	0.231533	−0.47177	0.070467	2.530333	−1.14687	1.383467
123LEU	1.357133	−0.0177	−0.11303	0.145	1.371367	−0.4431	0.928267
170ILE	0.658967	−0.00807	0.008033	0.053867	0.712833	−0.0471	0.665733
513ILE	0.9021	−0.02133	−0.08163	0.133067	0.932133	−0.33477	0.597367
105VAL	0.8276	−0.0239	−0.05133	0.039933	0.7923	−0.2665	0.5258
174LEU	0.583733	−0.0444	−0.04887	0.065467	0.5559	−0.05803	0.497867
209MET	0.815167	0.021433	−0.12723	0.117467	0.826833	−0.3365	0.490333
509THR	1.117	0.854933	−0.64543	0.1133	1.439733	−0.99017	0.449567
508VAL	0.763833	0.0468	−0.07763	0.067667	0.8007	−0.37477	0.425933
505TYR	0.535133	0.035733	−0.09663	0.0473	0.521567	−0.13563	0.385933
101VAL	0.5151	−0.0067	−0.02857	0.064667	0.5444	−0.20363	0.340767
177ILE	0.251267	−0.0026	−0.00083	0.0188	0.266633	−0.02537	0.241267
202ARG	0.238933	−0.06787	0.057833	0.0062	0.235	−0.01777	0.217233
160ILE	0.148267	−0.00497	0.0135	0.002933	0.1597	−0.00467	0.155033
120VAL	0.240267	−0.0054	0.029	−0.00053	0.263367	−0.13057	0.1328
200VAL	0.1169	−0.0056	0.013267	0.006967	0.131533	−0.00313	0.1284
111ASP	0.016433	0.062667	−0.04713	0	0.0318	−0.00083	0.030967
113GLU	0.026633	0.038833	−0.03197	0	0.0334	−0.00267	0.030733
166LYS	0.038567	−0.03273	0.025667	0	0.0314	−0.00277	0.028633
213TYR	0.028967	−0.00053	0.000433	3.33E−05	0.028933	−0.00043	0.0285
121GLU	0.019067	0.052167	−0.0439	0	0.027333	−0.00087	0.026467
TOTAL	16.02787	1.261767	−2.43747	1.2506	16.1021	−5.29773	10.80437

Table S19. Alanine scanning binding pocket within 5 Å of Lig13's binding free energy. All units are given in kcal/mol.

Mutation	dVDW	dEEL	dGB	dNP	dH	dIE_3S	dG
102VAL	1.6546	0.128067	−0.17323	0.1463	1.755833	−0.42337	1.332467
123LEU	1.6362	0.043933	−0.1751	0.151	1.656067	−0.33927	1.3168
116HIE	1.766133	0.037233	−0.2393	0.044067	1.6081	−0.3747	1.2334
164LEU	1.569933	−0.00543	−0.22217	0.1513	1.493733	−0.2781	1.215633
513ILE	1.354733	−0.01167	−0.0831	0.1258	1.385867	−0.24443	1.141433
105VAL	1.024267	0.008567	−0.07607	0.050567	1.0074	−0.15993	0.847467
509THR	0.9705	0.051667	−0.19077	0.074233	0.905633	−0.28103	0.6246
170ILE	0.609767	−0.00083	−0.0157	0.053067	0.6464	−0.02893	0.617467
97HIE	0.6324	0.043733	−0.10017	0.056833	0.632833	−0.09407	0.538767
101VAL	0.586733	−0.004	−0.08213	0.068933	0.5696	−0.1355	0.4341
174LEU	0.475333	−0.02913	−0.04737	0.048667	0.4476	−0.02737	0.420233
106ARG	0.493467	−0.05557	0.028533	0.0304	0.4968	−0.07787	0.418933
120VAL	0.408233	−0.0154	−0.0064	0.0075	0.394033	−0.07983	0.3142
508VAL	0.4644	−0.02423	−0.04397	0.030333	0.426633	−0.15377	0.272867
202ARG	0.2197	−0.02373	0.002133	0.012667	0.210867	−0.01967	0.1912
126LEU	0.160333	−0.0155	0.011967	0.011767	0.1686	−0.01003	0.158567
133PRO	0.128467	−0.01147	0.017433	0.0073	0.141767	−0.00523	0.136533
177ILE	0.115833	−0.00393	0.0073	0.001167	0.1204	−0.00267	0.117733
103PRO	0.064767	0.006767	0.004867	0.001133	0.077567	−0.00103	0.076533
165ASP	0.0285	−0.0088	0.012933	0.000133	0.032833	−0.0023	0.030533
TOTAL	14.3643	0.110267	−1.3703	1.073167	14.17857	−2.7391	11.43947

Table S20. Alanine scanning binding pocket within 5 Å of Lig14's binding free energy. All units are given in kcal/mol.

Mutation	dVDW	dEEL	dGB	dNP	dH	dIE_3S	dG
116HIE	1.864167	−0.13113	−0.10343	0.061833	1.6915	−0.418	1.2735
123LEU	1.538933	0.047667	−0.1417	0.144767	1.589733	−0.37057	1.219167
202ARG	1.280967	0.1403	−0.16453	0.136167	1.392867	−0.23363	1.159233
102VAL	1.583967	0.123467	−0.2033	0.149067	1.653367	−0.49957	1.1538
513ILE	1.413067	0.005333	−0.1604	0.141067	1.399033	−0.40337	0.995667
164LEU	1.267733	−0.01067	−0.1744	0.1261	1.208867	−0.23447	0.9744
105VAL	1.0776	0.0345	−0.06543	0.044467	1.0912	−0.21977	0.871433
170ILE	0.609633	0.004633	−0.00787	0.053367	0.659833	−0.04897	0.610867
509THR	0.816467	0.1046	−0.1166	0.042633	0.847233	−0.28643	0.5608
101VAL	0.666433	0.005167	−0.07893	0.0855	0.6783	−0.20153	0.476767
174LEU	0.4989	−0.0401	−0.03863	0.0453	0.4656	−0.02867	0.436933
106ARG	0.449033	−0.02407	0.0022	0.024	0.451167	−0.08003	0.371133
120VAL	0.383033	−0.03643	0.0305	0.003933	0.381033	−0.07217	0.308867
118ILE	0.166667	0.003467	0.074633	0.001167	0.2461	−0.01767	0.228433
133PRO	0.175933	−0.01253	0.0151	0.0144	0.193	−0.0294	0.1636
508VAL	0.443733	−0.03677	−0.05413	0.030833	0.383767	−0.2232	0.160567
103PRO	0.0694	0.002633	0.003367	0.001267	0.076733	−0.00113	0.0756
505TYR	0.0743	0.047867	−0.04793	0.000133	0.0745	−0.00657	0.067933
97HIE	0.083267	0.035467	−0.04847	0.0012	0.0716	−0.019	0.0526
209MET	0.072867	0.025867	−0.04023	0.0007	0.059267	−0.0123	0.046967
165ASP	0.029933	0.012233	−0.006	6.67E−05	0.0362	−0.00233	0.033867
TOTAL	14.56603	0.3015	−1.3262	1.107967	14.6509	−3.40877	11.24213

Table S21. Alanine scanning binding pocket within 5 Å of Lig15's binding free energy. All units are given in kcal/mol.

Mutation	dVDW	dEEL	dGB	dNP	dH	dIE_3S	dG
116HIE	1.827233	0.078733	−0.21217	0.046967	1.740833	−0.35447	1.386367
164LEU	1.286533	−0.00237	−0.05713	0.128667	1.355767	−0.2213	1.134467
105VAL	1.382567	0.055033	−0.21647	0.126833	1.348	−0.25517	1.092833
509THR	1.2974	0.059967	−0.1342	0.0648	1.288	−0.20333	1.084667
513ILE	1.1028	−0.00733	−0.06703	0.1042	1.1327	−0.14673	0.985967
123LEU	1.114567	0.023433	−0.06567	0.1127	1.185133	−0.2658	0.919333
202ARG	0.6531	0.121367	−0.14713	0.0628	0.690167	−0.10683	0.583333
170ILE	0.514133	−0.0005	0.0407	0.046267	0.600667	−0.0413	0.559367
101VAL	0.7346	0.0137	−0.10473	0.083633	0.7273	−0.19177	0.535533
209MET	0.702133	−0.01247	−0.10737	0.088633	0.671	−0.14367	0.527333
102VAL	0.8505	0.035467	−0.1608	0.082067	0.8073	−0.2873	0.52
120VAL	0.4536	−0.02063	0.0645	0.008233	0.5058	−0.08543	0.420367
508VAL	0.463567	−0.01127	0.015867	0.025367	0.493567	−0.08387	0.4097
174LEU	0.2956	−0.0159	0.0199	0.020533	0.3202	−0.01147	0.308733
510TYR	0.209733	−0.02263	0.0371	0.000233	0.2245	−0.0094	0.2151
200VAL	0.146633	−0.0122	0.0235	0.007867	0.165867	−0.0047	0.161167
505TYR	0.095167	−0.0267	0.024433	0.001467	0.094467	−0.0041	0.090367
133PRO	0.072867	−0.01223	0.0145	0.001967	0.0771	−0.00223	0.074867
97HIE	0.066833	0.030467	−0.04763	0	0.0498	−0.00323	0.046567
113GLU	0.0461	0.0469	−0.04687	0	0.046167	−0.00217	0.044
165ASP	0.0261	0.077633	−0.05967	6.67E−05	0.044167	−0.00097	0.0432
TOTAL	13.34177	0.398467	−1.18637	1.0133	13.5685	−2.42523	11.14327

Table S22. Result of In vitro enzyme activity assays.

Name	dH	dIE_3S	dG	Docking Score	Shape Sim	IC_{50}	Resource
CHEMBL1514025	22.0281	−4.2868	17.7413	−10.044	0.151	*	receptor based
ZINC000009641177	21.3661	−3.6825	17.6836	−10.836	0.236	*	receptor based
ZINC000067300707	21.5464	−3.9062	17.6402	−11.357	0.197	*	receptor based
ZINC000009640749	20.2565	−2.8168	17.4397	−10.941	0.122	>100	receptor based
ZINC000002691260	20.607	−3.4168	17.1902	−10.808	0.236	*	receptor based
ZINC000067301214	21.4859	−4.3381	17.1478	−11.17	0.148	*	receptor based
ZINC000067301018	21.2853	−4.1764	17.1089	−10.947	0.277	>100	receptor based
ZINC000067300621	21.0244	−3.977	17.0474	−10.996	0.172	*	receptor based
CHEMBL1509241	21.5991	−4.6284	16.9708	−10.184	0.092	*	receptor based
ZINC67300323	19.7742	−3.398	16.3762	−10.657	0.191	*	receptor based
CHEMBL468176	20.0583	−1.4102	18.6481	−8.743	0.144	16.86 μM	ligand-based 3d
ZINC20897666	22.6537	−4.8417	17.812	−8.268	0.197	57.33 μM	ligand-based 3d
ZINC000329326969	20.4952	−2.9187	17.5765	−8.569	0.238	*	ligand-based3d

*: The oxidation is too strong to calculate the reaction rate.
